# Role of Molecular Modification and Protein Folding in the Nucleation and Growth of Protein–Metal–Organic Frameworks

**DOI:** 10.1021/acs.chemmater.2c01903

**Published:** 2022-09-15

**Authors:** Brooke P. Carpenter, A. Rain Talosig, Justin T. Mulvey, Jovany G. Merham, Jamie Esquivel, Ben Rose, Alana F. Ogata, Dmitry A. Fishman, Joseph P. Patterson

**Affiliations:** † Department of Chemistry, University of California Irvine, Irvine, California 92697-2025, United States; ‡ Department of Materials Science and Engineering, University of California Irvine, Irvine, California 92697-2025, United States

## Abstract

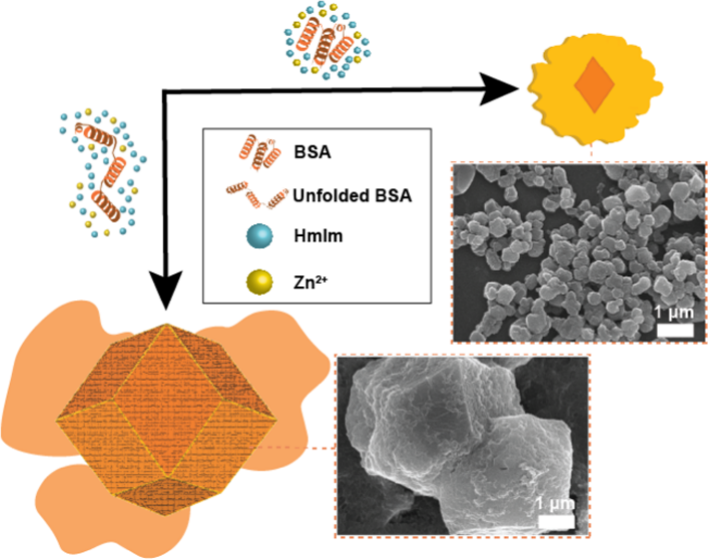

Metal–organic frameworks (MOFs) are a class of porous nanomaterials that have been extensively studied as enzyme immobilization substrates. During in situ immobilization, MOF nucleation is driven by biomolecules with low isoelectric points. Investigation of how biomolecules control MOF self-assembly mechanisms on the molecular level is key to designing nanomaterials with desired physical and chemical properties. Here, we demonstrate how molecular modifications of bovine serum albumin (BSA) with fluorescein isothiocyanate (FITC) can affect MOF crystal size, morphology, and encapsulation efficiency. Final crystal properties are characterized using scanning electron microscopy (SEM), powder X-ray diffraction (PXRD), fluorescent microscopy, and fluorescence spectroscopy. To probe MOF self-assembly, in situ experiments were performed using cryogenic transmission electron microscopy (cryo-TEM) and X-ray diffraction (XRD). Biophysical characterization of BSA and FITC-BSA was performed using ζ potential, mass spectrometry, circular dichroism studies, fluorescence spectroscopy, and Fourier transform infrared (FTIR) spectroscopy. The combined data reveal that protein folding and stability within amorphous precursors are contributing factors in the rate, extent, and mechanism of crystallization. Thus, our results suggest molecular modifications as promising methods for fine-tuning protein@MOFs’ nucleation and growth.

## Introduction

Living systems have evolved enzymes to have remarkable catalytic efficiency and stereoselectivity.^1^ However, most enzymes evolved in specific environments that did not promote enzymes with high thermal or chemical stability. Consequently, the implementation of enzymes into industrial applications, which are typically performed in nonphysiological environments, has been limited by enzyme stability.^2,3^ Enormous efforts have been devoted to enhancing the stability and recyclability of enzymes by immobilizing them onto supporting structures to significantly reduce the energy and economic cost of the chemical industry.^4,5^ One promising support strategy utilizes metal–organic frameworks (MOFs) due to their large surface area, simplistic synthetic conditions, and tunable pore and crystal sizes.^5−9^ MOFs consist of coordinated metal ions and organic ligands building blocks that form protective frameworks for biomolecules.^10−12^

Enzymes can be integrated into MOF systems through in situ approaches in which the crystal forms in the presence of a protein,^6,7^ or post-synthetic approaches, where the protein is incorporated after crystallization by surface attachment,^13^ pore entrapment,^14^ or covalent linkage.^15,16^ In situ approaches are advantageous due to their mild synthetic conditions, simplistic synthetic procedures, and typically higher encapsulation efficiencies (EE%).^7,17^ The primary challenge with the in situ approach is understanding how the biomolecules affect the nucleation and growth of the MOF crystals and become incorporated into frameworks. Low isoelectric point (pI) (<7) proteins have been shown to effectively initiate the nucleation of zeolitic imidazole framework-8 (ZIF-8) when precursors are below supersaturation conditions.^18^ High pI (>7) proteins cannot initiate nucleation, but molecular modifications of proteins can be used to lower the pI and promote nucleation.^18^ However, the role molecular modification and protein folding play in controlling crystal properties such as size and morphology has not been established. These properties are essential for the catalytic performance of protein@MOFs as they determine the accessibility of enzymes to substrates. For example, when an enzyme is located throughout the crystal, smaller crystal sizes are desired to reduce the diffusion barrier and allow the substrate to reach the internal enzymes.^19^ Furthermore, a recent study demonstrated that variation in the protein@MOF crystal structure directly affects enzymatic activity, which supports the need for understanding nucleation and growth mechanisms to optimize protein@MOFs’ properties.^20^

Here, we demonstrate how molecular modification of a protein affects encapsulation efficiency, crystal size, and morphology of protein@MOFs. One of the most common molecular modifications for proteins in MOF systems is a fluorescent tag such as fluorescein isothiocyanate (FITC), which aids in determining the encapsulation efficiency and location of the protein in a crystal.^6,17^ In our studies, bovine serum albumin (BSA) and FITC-BSA are used as model proteins as they have been well studied and are inexpensive.^21−23^ Biophysical characterization of the proteins was performed using mass spectrometry, circular dichroism, and ζ potential measurements. To compare final protein@MOF crystals, scanning electron microscopy (SEM), transmission electron microscopy (TEM), fluorescent microscopy, powder X-ray diffraction (PXRD), and fluorescence spectrometry are used. Encapsulation efficiencies of BSA and FITC-BSA are determined using a developed procedure to measure intrinsic tryptophan fluorescence by accounting for potential interaction between protein and MOF precursors. To evaluate how the FITC modification affects the nucleation and growth mechanism, cryogenic TEM (cryo-TEM) and in situ XRD are performed.

## Results

### BSA@ZIF-8 and FITC-BSA@ZIF-8 Syntheses

Stock solutions of 2-methylimidazole (HmIm) (5600 mM, 2800 mM, 1400 mM, 700 mM, and 320 mM, 0.5 mL), zinc acetate (40 mM, 1 mL), and protein (10 mg/mL, 5 mg/mL, and 2.5 mg/mL, 0.5 mL) were prepared with Milli-Q water (18 MΩ). Stock solutions were used to prepare a series of MOF crystallization experiments with variation in the HmIm/Zn ratio (70:1, 35:1, 17.5:1, 4:1) and protein concentration (2.5, 1.25, and 0.625 mg/mL) (Table 1). Protein solutions were added to 2-methylimidazole solutions, and crystallization was initiated by the addition of a zinc acetate solution. Solutions were aged for 24 h without stirring. The precipitate was obtained via centrifugation at 10,000 rpm for 10 min, where the supernatant was kept for EE% measurements.^6,24,25^ Precipitates were then washed with water three times prior to electron microscopy and PXRD analysis.

**Table 1:** Summary of Crystal Sizes for BSA@ZIF-8 and FITC-BSA@ZIF-8 at Four Different HmIm/Zn Ratios (4:1, 17.5:1, 35:1, 70:1) with Final Protein Concentrations of 2.5, 1.25, and 0.625 mg/mL

			crystal size (nm)	
HmIm/Zn (mM:mM)	ratio HmIm/Zn	final protein concentrations (mg/mL)	BSA	FITC-BSA
80:20	4:1	2.5	184 ± 31	944 ± 197
		1.25	187 ± 45	1317 ± 214
		0.625	229 ± 41	2065 ± 282
700:20	17.5:1	2.5	203 ± 42	2215 ± 391
		1.25	296 ± 63	403 ± 51
		0.625	292 ± 94	389 ± 52
1400:20	35:1	2.5	228 ± 57	1183 ± 334
		1.25	270 ± 50	585 ± 189
		0.625	291 ± 40	1281 ± 301
2800:20	70:1	2.5	215 ± 33	486 ± 212
		1.25	229 ± 49	402 ± 51
		0.625	316 ± 61	389 ± 51

### Protein Characterization

**Figure 1:**
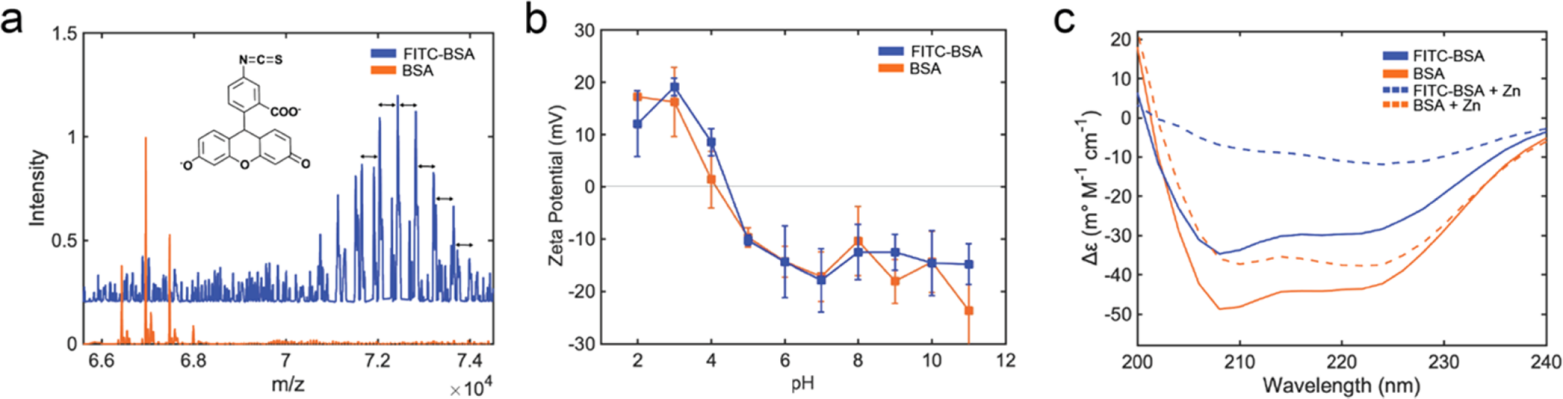
Biophysical characterization of FITC-BSA (blue) and BSA (orange) using (a) ESI-MS where the black arrows are indicative of FITC-tag spacing, (b) ζ potential, and (c) circular dichroism in the absence (solid line) and presence (dashed line) of zinc.

BSA and FITC-BSA underwent biophysical characterization using mass spectroscopy techniques, circular dichroism, and ζ potential measurements. Mass spectra of FITC-BSA indicate heterogeneous FITC tagging by the poor signal-to-noise in the raw spectra and the multiple peaks found in the deconvoluted spectra (Figure 1a). Charge state deconvolution was performed on the data, and the center of mass for BSA was determined to be 66,955 g/mol, which aligns with the reported mass in the literature,^26^ and the center of mass for FITC-BSA to be 72,433 g/mol. FITC-BSA was found to have 12–18 FITC tags per biomolecule. To determine how FITC affects surface charge, ζ potential measurements were performed on BSA and FITC-BSA at pH ranges from 2 to 11 (Figure 1b). Measurements revealed that both proteins have similar isoelectric points (∼4–4.5) and that both were highly negatively charged in the pH conditions that occur during MOF synthesis. Circular dichroism was used to measure the secondary protein structure of the tagged and untagged protein in the absence and presence of zinc acetate to understand how ZIF-8 precursors affect the protein structures (Figure 1c). All samples were performed at the same protein concentration of 1 mg/mL. Studies were attempted in the presence of HmIm, but the quantum yield of HmIm was too high for the instrument detector as HmIm absorbs in the UV wavelength range. However, HmIm is believed to also affect protein folding.^27^ BSA is a globular protein that consists of predominantly α-helical content. The CD band for α-helical proteins has characteristic peak dips at ∼210 and ∼220 nm.^28^ A reduction in ellipticity (Δε) at these peak dips is representative of protein unfolding. It was found that the α-helical character of BSA was reduced when modified with FITC or when in the presence of zinc acetate. When FITC-BSA is in the presence of zinc acetate, the α-helical character significantly decreases compared to all other samples. Intrinsic tryptophan fluorescence further confirms the unfolding of BSA when tagged with FITC (Figure S1). A blue shift can be observed for FITC-BSA compared to BSA as the center of mass changes from 345 to 310 nm. While we also see a blue shift (∼5–10 nm) for BSA@ZIF-8, the shift is more significant for FITC-BSA, where the center of mass shifts from 310 to 380 nm. We can associate these changes with structural changes of protein molecules that influence the position of energy states as well as transition probability.^29^ While protein unfolding has been shown to occur in the presence of zinc acetate and when encapsulated in ZIF-8, refolding of the protein upon release from ZIF-8 is possible.^30^

### Crystal Structure

**Figure 2:**
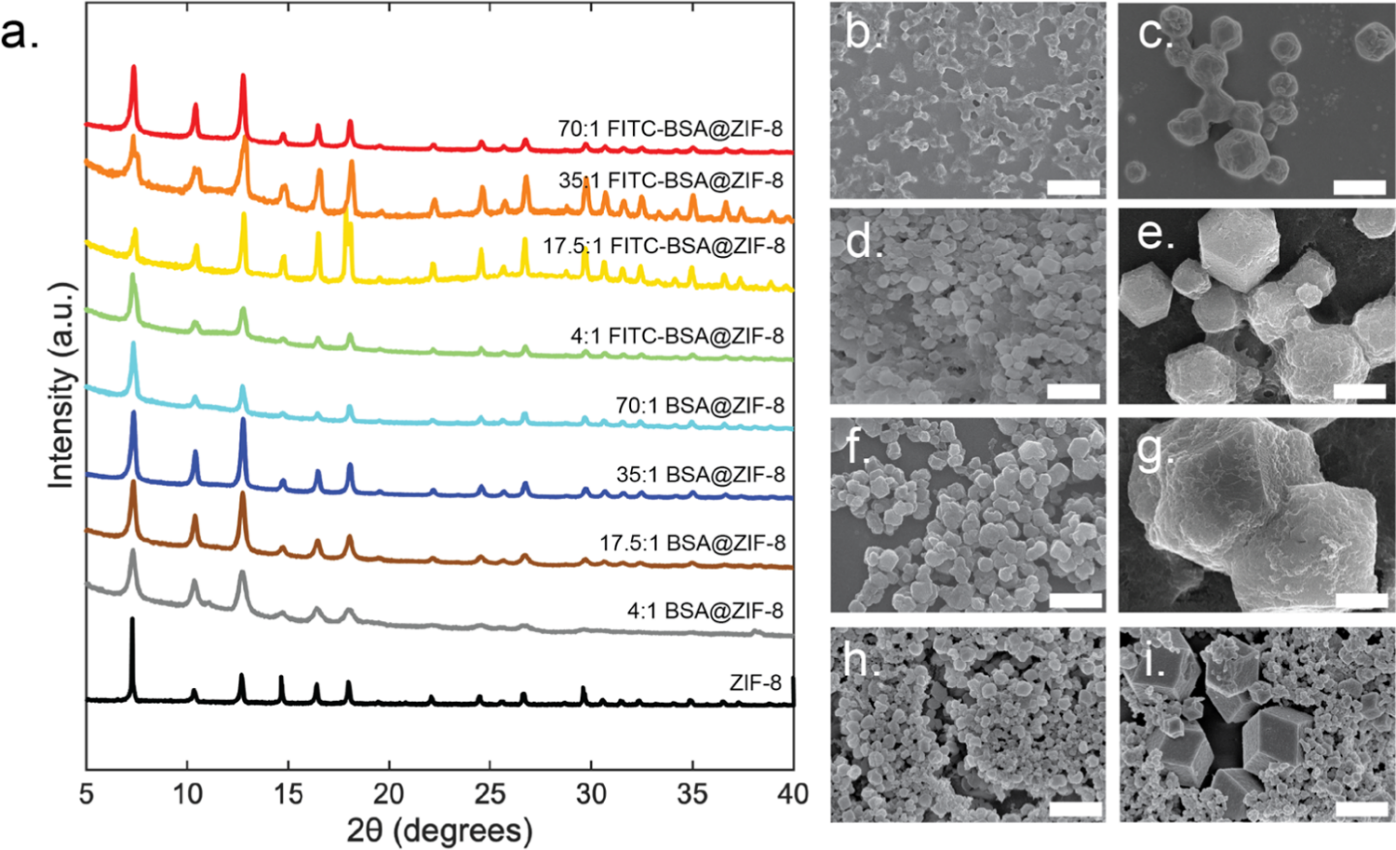
(a) PXRD patterns of protein@MOFs at varying HmIm ratios. SEM images of (b) 4:1 BSA@ZIF-8, (c) 4:1 FITC-BSA@ZIF-8, (d), 17.5:1 BSA@ZIF-8, (e) 17.5:1 FITC-BSA@ZIF-8, (f) 35:1 BSA@ZIF-8, (g) 35:1 FITC-BSA@ZIF-8 (h) 70:1 BSA@ZIF-8, and (i) 70:1 FITC-BSA@ZIF-8, at a final protein concentration of 2.5 mg/mL. The white scale bar is at 1 μm.

ZIF-8 crystals can form various polymorphs, with diamondoid (dia) and sodalite (sod) being the most widely studied.^31,32^ Polymorph control can be obtained by altering the HmIm/Zn ratio, changing the precursor concentrations, or integrating a nucleation and growth-driving agent (i.e., surfaces or biomolecules).^33,34^ PXRD was used to analyze isolated ZIF-8, BSA@ZIF-8, and FITC-BSA@ZIF-8 crystals. At low HmIm/Zn ratios (4:1), ZIF-8 crystals form the diamondoid (dia) structure (Figure S2a).^35^ The HmIm/Zn ratio gradually increases (17.5:1), and a mixture of dia and sod can be obtained (Figure S2b), followed by exclusive sod formation at 35:1 and 70:1 (Figure S2c,d). The sod polymorph is also formed exclusively for all HmIm/Zn ratios except 4:1 in the presence of BSA and FITC-BSA (Figures 2, S3, and S4).^21^ For 1.25 and 0.625 mg/mL at 4:1, a mixture of sod and ZIF-CO_3_-1 (ZIF-C) can be observed for both protein@MOFs (Figures S3a ad S4a), This is not surprising as it has been recently found that ZIF-C forms as the weight percent of BSA decreases.^22^

### Crystal Size

**Figure 3:**
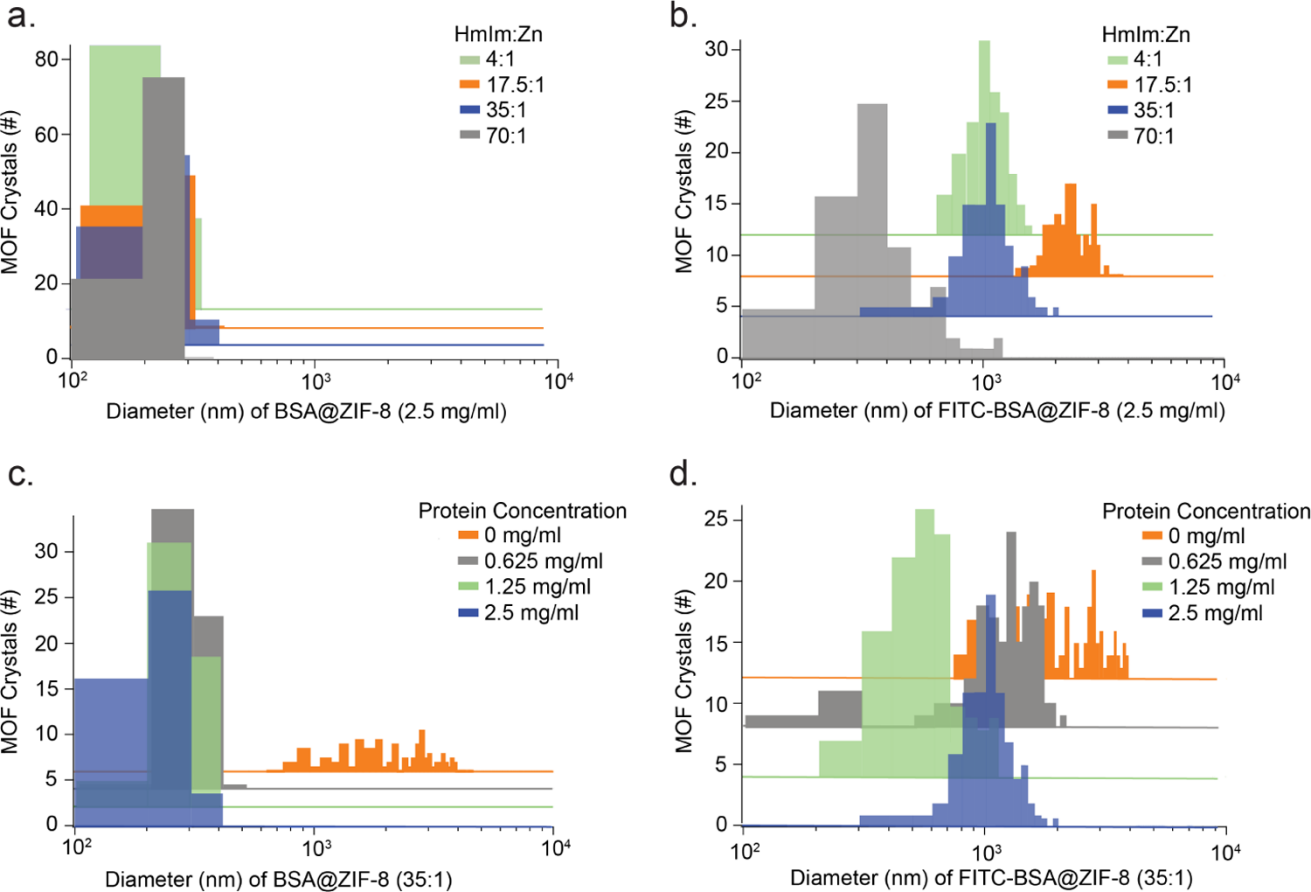
Size distribution histograms of (a) BSA@ZIF-8 and (b) BSA@ZIF-8 at a constant protein concentration of 2.5 mg/mL and HmIm:zinc ratios of 4:1 (green), 17.5:1 (orange), 35:1 (blue), and 70:1 (gray) and (c) BSA@ZIF-8 and (d) FITC-BSA at a constant HmIm:zinc ratio of 35:1 with final protein concentrations of 2.5 mg/mL (blue), 1.25 mg/mL (green), 0.625 mg/mL (gray), and 0 mg/mL (orange).

ZIF-8 (sod) crystals form large crystals with a large particle size distribution. For example, with 35:1, crystal sizes range from 710 nm to 3.7 μm with an average mean diameter of 2.1 μm and an average standard deviation of ∼800 nm (Figure 3). By integrating BSA into a ZIF-8 system, the crystal size and standard deviation decrease with an average crystal size of 245 nm and an average standard deviation of 50 nm. At all HmIm/Zn conditions, the crystal size gradually decreases as the BSA concentration increases (Figures 3c and S6a). For example, with 4:1, the average crystal size with 0.625 mg/mL BSA is 229 nm, and the average crystal size with 2.5 mg/mL BSA is 184 nm. For all synthetic conditions with FITC-BSA, the average mean size of crystals (1.3 μm) is larger than BSA@ZIF-8 crystals (245 nm) but smaller than ZIF-8 crystals (1.4 μm) (Table 1). In addition, the average standard deviation for FITC-BSA is 194 nm, which is greater than the average standard deviation of BSA@ZIF-8 (50 nm) but smaller than that of ZIF-8 (800 nm). Except for the 4:1 condition, size trends related to protein concentration or HmIm/Zn cannot be observed for FITC-BSA@ZIF-8 (Figures 3b,d and S6b).

### Crystal Morphology

In the absence of protein, ZIF-8 crystals exhibit smooth surfaces for both sod and dia polymorphs (Figure S2). Introduction of BSA at all HmIm/Zn ratios results in the formation of spheroid crystals with rough surfaces (Figure 2b,d,f,h). As the ratio of HmIm/Zn increases, BSA@ZIF-8 crystals become more faceted and have smoother surfaces (Figure S3). In the presence of FITC-BSA, crystals retain truncated rhombic dodecahedral morphology, displaying rough surfaces at low HmIm/Zn ratios (Figure 2c,e) and smoother surfaces at high ratios (Figure 2g,i). The 70:1 FITC-BSA@ZIF-8 crystals form three different types of crystals that can be described as large smooth-surfaced crystals, mid-sized crystals with rougher surfaces, and small spheroid crystals with rough surfaces (Figure 2i). All MOF crystals were washed three times with water to remove excess precursors, yet significant amorphous peaks in PXRD patterns can be observed in the 17.5:1 with 1.25 mg/mL of BSA as well as with 17.5:1 and 35:1 with 2.5 mg/mL FITC (Figure 2a). A comparison of BSA@ZIF-8 and FITC-BSA@ZIF-8 crystals (35:1, 2.5 mg/mL) by TEM indicates that the FITC-BSA@ZIF-8 sample contains large regions of undefined material (Figure S8). We hypothesize that this undefined material contributes to the amorphous peak seen in PXRD.

### Encapsulation Efficiency

**Figure 4:**
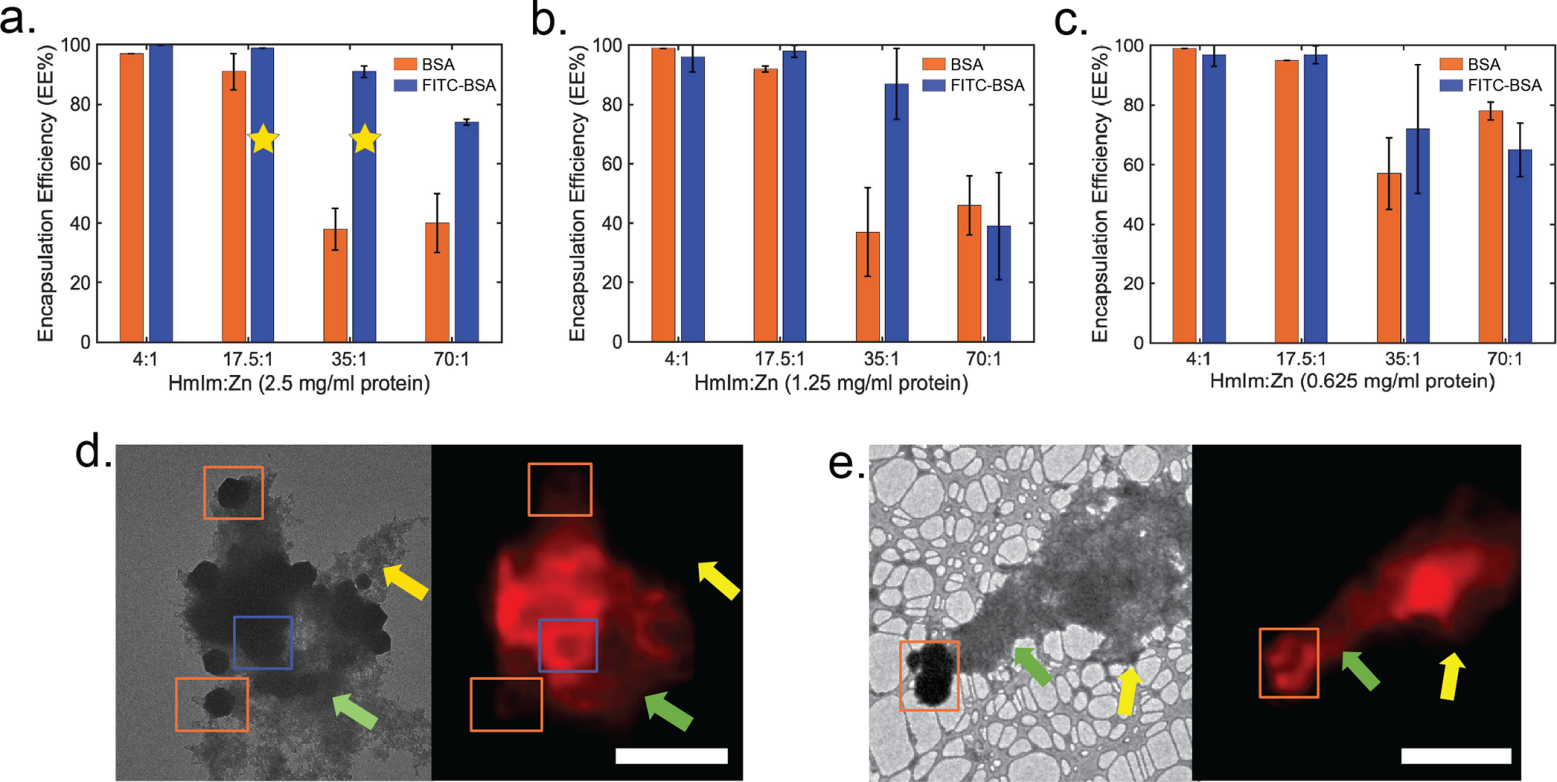
Encapsulation efficiency of protein@MOFs at final protein concentrations of (a) 2.5 mg/mL, (b) 1.25 mg/mL, and (c) 0.625 mg/mL. (d, e) Correlative TEM (left) and fluorescence microscopy (right) images of FITC-BSA@ZIF-8 crystals at 35:1 with 2.5 mg/mL FITC-BSA. Scale bars are 10 μm. A strong, uniform signal can be viewed around the outside edges of some crystals (blue box), whereas a weak, nonuniform signal can be seen in other crystals (orange box). Green arrows denote an amorphous phase with a clear fluorescent signal, whereas the yellow arrow denotes an amorphous region that has little to no fluorescence.

Protein incorporation into BSA@ZIF-8 and FITC-BSA@ZIF-8 was confirmed with FTIR spectra as amide I peak at 1654 cm^–1^ can be observed in both MOF samples (Figure S9).^36,37^ Additionally, intrinsic tryptophan fluorescence was performed on the protein@MOFs (Figure S1). For both protein@MOFs, samples were excited at 280 nm, and emission peaks can be observed between 310 and 380 nm, which can be linked to tryptophan amino acids, and thus protein, being incorporated into the MOFs. The encapsulation efficiency for FITC-BSA@ZIF-8 and BSA@ZIF-8 was determined by measuring the concentration of protein in the supernatant,^6,17,18^ which is the liquid obtained after the first centrifugation cycle prior to washes. EE% is calculated by quantifying the remaining protein concentration in the supernatant to calculate protein concentration in MOF precipitate. EE% was measured using fluorescence spectroscopy, where the emission intensity of fluorescein (∼520 nm) and tryptophan (∼340 nm) was measured for FITC-BSA@ZIF-8 and BSA@ZIF-8, respectively (Figure 4). Tryptophan fluorescence intensity is sensitive to solution pH and metal binding; thus, supernatants for BSA@ZIF-8 and FITC-BSA@ZIF-8 were diluted in a phosphate buffer (∼pH 6.7) containing excess tetrasodium ethylenediaminetetraacetic acid (EDTA) to ensure that the protein conformation remained constant. (Figure S11).

Protein EE% measurements of BSA@ZIF-8 systems were also measured using the Bradford assay, which validated results from the fluorescent measurements (Figure S14). When HmIm/Zn ratios of 4:1 or 17.5:1 are used, both BSA@ZIF-8 and FITC-BSA@ZIF-8 have ∼100% encapsulation for all protein concentrations studied. The most significant difference was observed in the 35:1 samples at protein concentrations of 2.5 and 1.25 mg/mL, where the EE% of FITC-BSA@ZIF-8 was ∼90% and the BSA@ZIF-8 was ∼40%. The EE% method for these results is notable because EE% is measured through protein quantification of the supernatant; therefore, the protein concentration in the MOF precipitate, which could potentially contain both MOF crystals and MOF amorphous phases, is the calculated EE% value. The 35:1 crystals were then imaged with fluorescent microscopy, which revealed that FITC-BSA is located in both crystals and the amorphous material (Figure 4d,e). Crystals with little to no fluorescence can also be observed in the fluorescent microscopy data. Thus, samples that contain a significant amorphous background, as indicated by PXRD, are indicated with a star. Future work will focus on methods to distinguish between proteins that are encapsulated into crystals and that are precipitated into an amorphous phase.

### Mechanistic Studies

**Figure 5:**
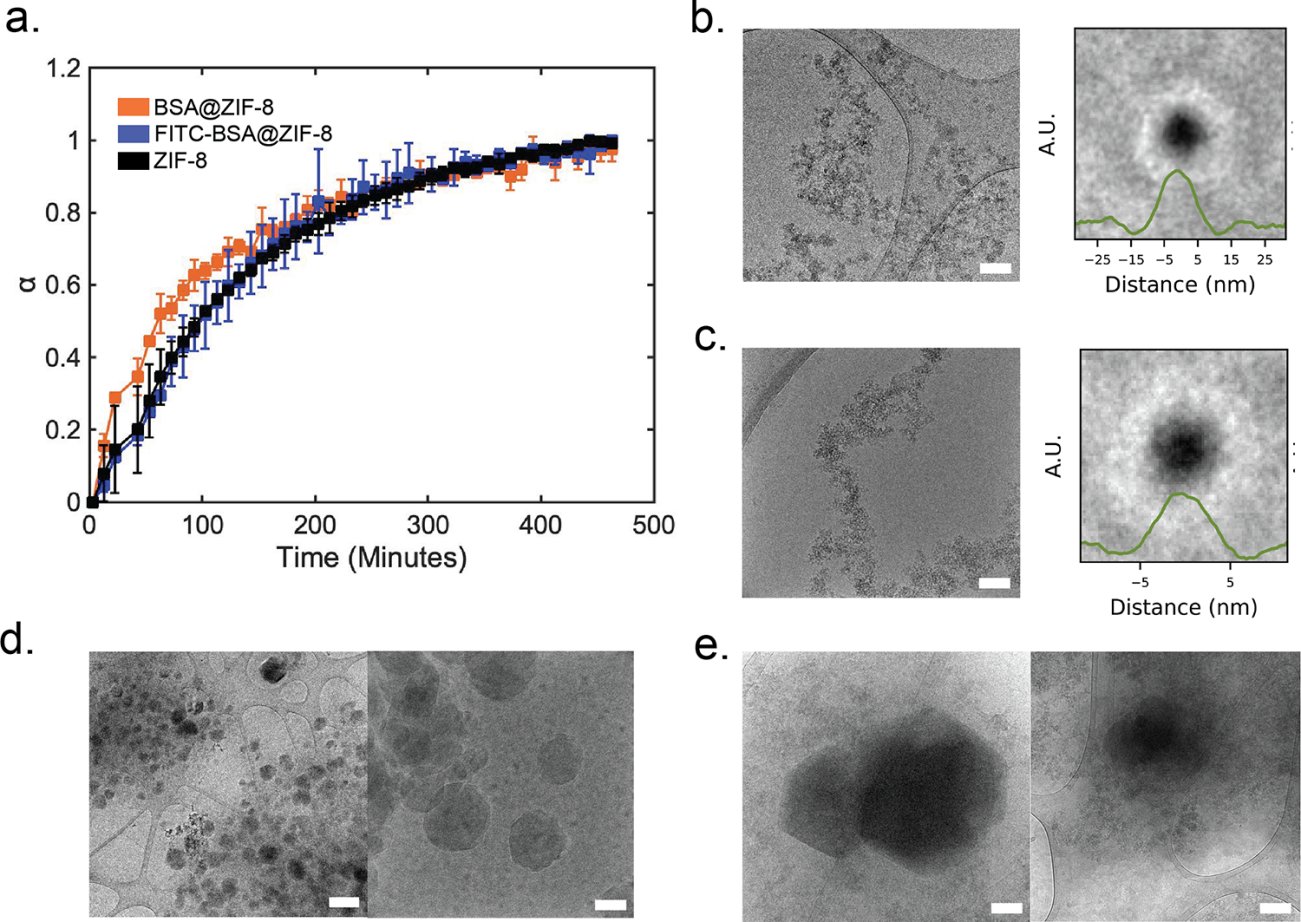
In situ measurements of protein@MOFs. (a) In situ XRD of BSA@ZIF-8 (orange), FITC-BSA@ZIF-8 (blue), and ZIF-8 (black). The data are displayed as the extent of crystallinity (α) over time. (b) Cryo-TEM image of FITC-BSA@ZIF-8 at 1 min (scale bar 100 nm) and averaged particles (right). (c) Cryo-TEM image of BSA@ZIF-8 at 1 min (scale bar 100 nm) (left) and averaged particles (right). Note: the data used for Figure 5c were collected in our previous paper.^21^ (d) Low-magnification cryo-TEM image (scale bar 1 μm) (left) and the high-magnification image (scale bar 100 nm) (right) of BSA@ZIF-8 at 1 h. (e.) Cryo-TEM of FITC-BSA@ZIF-8 at 1 h showing the appearance of the crystal (left) and amorphous particles (right). The scale bar is 100 nm.

In situ measurements were performed on the crystallization of FITC-BSA@ZIF-8 using XRD and cryo-TEM. Based on the data in the previous sections, the 35:1 BSA@ZIF-8 and FITC-BSA@ZIF-8 with a protein concentration of 2.5 mg/mL were chosen to study as the systems having the largest variance in crystal size, morphology, and encapsulation efficiency. In situ XRD data were analyzed by measuring the area under the (011) peak over 8 h to measure the extent of crystallinity (Figure 5a). As the reaction progresses, more amorphous species crystallize, which can be quantified by an increase in area under the (011) peak (Figure S15). Distinct differences between the growth of BSA@ZIF-8 and FITC-BSA@ZIF-8 can be observed between 10 and 110 min. During this time, BSA@ZIF-8 is observed to have a greater extent of crystallinity and to grow at a greater crystallization rate. ZIF-8 in the absence of protein was also measured and crystallized at a similar rate as FITC-BSA@ZIF-8. Time-point measurements for cryo-TEM were chosen based on differences in the in situ XRD data. At initial time points (∼1 min), all cryo-TEM images of FITC-BSA@ZIF-8 reveal similar Zn/HmIm amorphous and protein/Zn/HmIm amorphous phases (Figure 5b). Particle picking and averaging of individual particles were used to determine average particle diameters (see the SI for details). The full width at half maximum (FWHM) of the BSA@ZIF-8 particle line profile was 6.8 ± 1 nm (Figure 5c) and the FITC-BSA@ZIF-8 particle line profile was 12.0 ± 0.55 nm (Figure 5b). Note: the data used for Figure 5a were collected in our previous paper.^21^ After 1 h, the particulate amorphous phase disappears for BSA@ZIF-8 and is replaced with predominantly BSA@ZIF-8 crystals (Figure 5d). Meanwhile, at 1 h, the FITC-BSA@ZIF-8 sample still contains the particulate amorphous phase, which is either in the presence or absence of crystals (Figure 5e). Particle picking and averaging were attempted for the FITC-BSA@ZIF-8 amorphous particles at 1 h. However, the resulting image did not reveal a well-defined particle, which we believe is due to the heterogeneity of the particles within the amorphous phase (Figure S16). After 24 h, the BSA@ZIF-8 sample solely consists of crystals, whereas particulate amorphous phases can still be observed in the FITC-BSA@ZIF-8 samples (Figure S8b).

## Discussion

Previous research has demonstrated that BSA can be incorporated into ZIF-8 crystals via two different mechanisms that are dependent on HmIm/Zn ratios.^21^ At low ratios, BSA binds with Zn and HmIm, forming an amorphous precursor phase, which increases local supersaturation and promotes nucleation of ZIF-8 (sod). At high ratios, ZIF-8 crystals can form independently, and BSA is incorporated when amorphous particles of BSA/HmIm/Zn attach to the surface of growing crystals and undergo crystallization by particle attachment. The mechanism of particle attachment results in rough surfaces observed for BSA@ZIF-8 crystals. Although these are described as separate mechanisms, both mechanisms likely occur simultaneously under certain conditions. With this understanding, this paper aims to determine how molecular modifications affect the mechanisms through observation of in situ experiments and final crystal sizes and morphologies.

**Figure 6:**
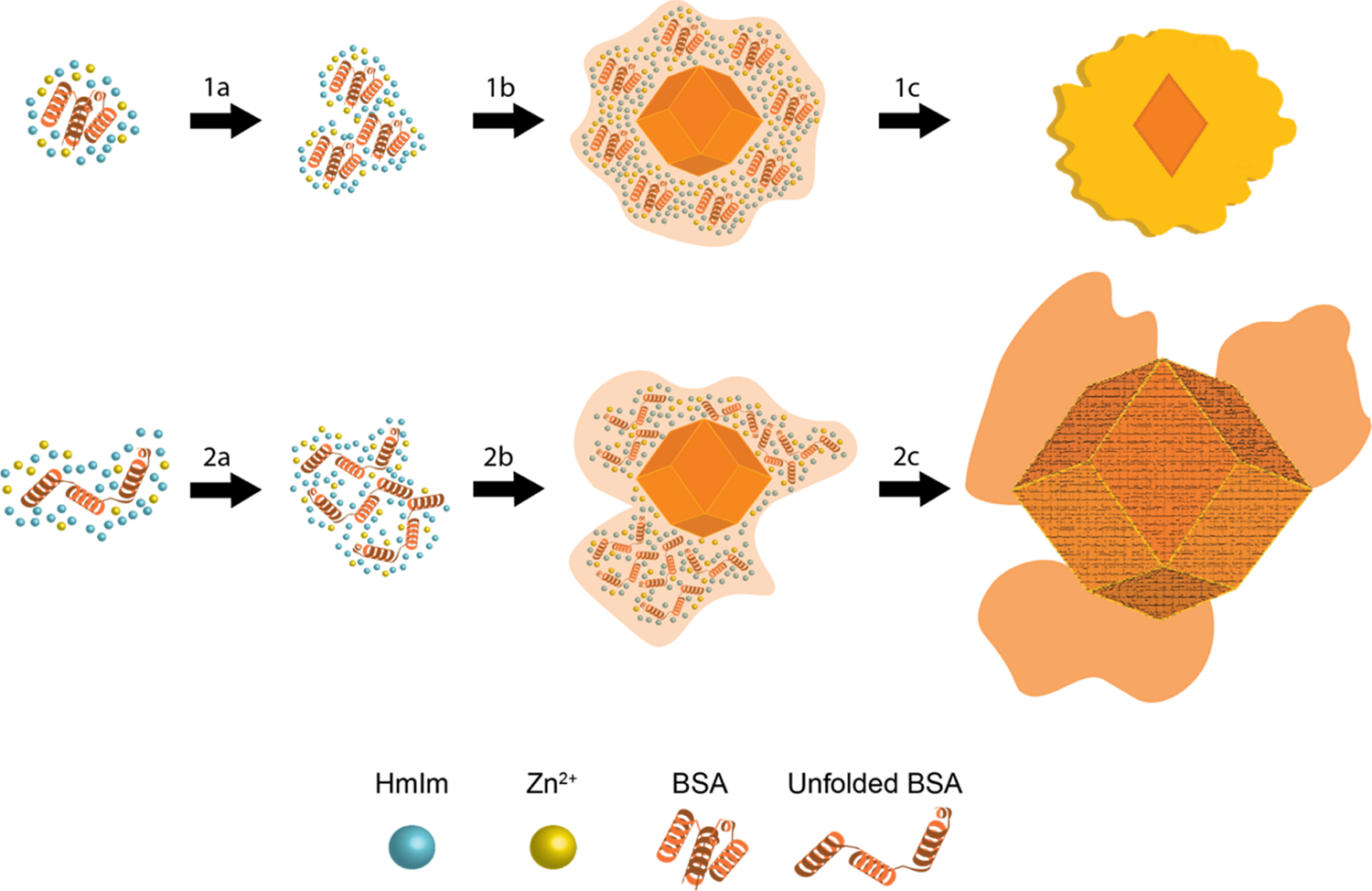
Proposed schematic of the formation mechanism of BSA@ZIF-8 at high HmIm/Zn ratios when BSA is (1a–1c) folded vs (2a–2c) unfolded. Both mechanisms initially form particulate amorphous intermediates consisting of protein/HmIm/Zn (a, b). The folded BSA/HmIm/Zn intermediates are then able to adsorb and crystallize onto the growing ZIF-8 surface (1c), whereas the unfolded BSA/HmIm/Zn intermediate cannot (2c).

In the case where proteins directly promote nucleation (low HmIm/Zn ratios), large mean crystal sizes and large size distributions indicate that nucleation from the protein/HmIm/Zn amorphous phase is slower with FITC-BSA@ZIF-8 than BSA@ZIF-8 (Figure 3, Table 1). When proteins are incorporated by particle attachment (high HmIm/Zn ratios), the collective data indicate that FITC-BSA can readily form an amorphous phase with HmIm and Zn (Figure 6(2a,2b)). Moreover, the larger FITC-BSA@ZIF-8 crystals (Table 1) indicate a slower rate of particle nucleation on the surface of growing ZIF-8 crystals (Figure 6(2c)). The SEM images further support this as FITC-BSA@ZIF-8 crystals have smoother surfaces compared to the BSA@ZIF-8 crystals (Figure 2).

EE% provides information on how efficiently particular growth mechanisms incorporate biomolecules into the final MOF products. At low HmIm/Zn ratios, EE% measurements suggest both FITC-BSA and BSA are successful in promoting ZIF-8 (sod) growth as both are fully incorporated into the MOF crystals. At high HmIm/Zn ratios (70:1 and 35:1), the EE% measurements suggest that FITC modification can increase the encapsulation of BSA into ZIF-8. However, the XRD and fluorescent microscopy data show that FITC-BSA@ZIF-8 products consist of both amorphous and crystalline phases, in contrast to BSA@ZIF-8 products, which almost exclusively consist of crystalline phases (Figures 2 and 4). This also supports our hypothesis that nucleation from the amorphous phase is inhibited by FITC-BSA.

To further test this hypothesis, in situ XRD and cryo-TEM measurements on the 35:1 protein@MOF at a 2.5 mg/mL protein concentration were performed to understand how FITC-BSA mechanistically alters the final MOF crystal properties and phases. The cryo-TEM data show that particulate amorphous phase forms for both BSA@ZIF-8 and FITC-BSA within 1 min. Some of the particles within the phases appear to consist of a protein core and a HmIm/Zn shell (Figure 5a). We further believe that HmIm and Zn are bound throughout the protein cores and that the zinc-bound atoms are responsible for the dark contrast of the particles. These FITC-BSA/HmIm/Zn particles persist for the remaining duration of the FITC-BSA@ZIF-8 synthesis, as supported by the cryo-TEM, whereas the BSA/HmIm/Zn particles adsorb and crystallize onto a growing ZIF-8 crystal. In addition, the rate of crystallinity development for FITC-BSA@ZIF-8 in the 10–100 min is slow compared to BSA@ZIF-8. This provides direct evidence for the inability for the FITC-BSA/HmIm/Zn particles to grow by particle attachment. Instead, this suggests that a monomer addition mechanism is favored.^21^

The collective data strongly support that FITC-BSA has a different nucleation and growth mechanism compared to BSA. Despite the size of fluorescent dyes being relatively small in comparison to proteins, research has found that fluorescent tags such as FITC can affect physicochemical characteristics of biomolecules such as size, secondary protein structure, and surface charge.^31,32^ Our initial hypothesis was that protein charge would be the main factor enabling particle addition crystallization.^18,38^ However, ζ potential measurements revealed similar pI (±0.5) for FITC-BSA and BSA (Figure 1b). Instead, circular dichroism studies suggest variation in protein folding, especially within the amorphous precursor phase, to be the cause of the deviation in protein@MOF formation mechanisms. In general, protein unfolding causes hydrophobic amino acid groups to become exposed and protein aggregation to occur.^39^ In the case of FITC-BSA@ZIF-8 and BSA@ZIF-8, we hypothesize that unfolding is caused by the high binding affinity of zinc to the electrophilic groups on the amino acids and FITC tag. While the unfolding of BSA is minimal in the presence of zinc, as supported by the recent literature,^40^ FITC tagging increases the extent of the unfolding of BSA with zinc (Figure 1c). The unfolding of FITC-BSA in the amorphous phase explains the large heterogeneity of amorphous particles at 1 h (Figure S16). To further validate the effect of protein unfolding in the growth mechanisms, BSA@ZIF-8 crystals were synthesized using a partially unfolded BSA (Figure S17). Crystals produced with partially unfolded BSA had a similar morphology as FITC-BSA@ZIF-8 crystals (Figure S17b) and were ∼800 nm—much larger than BSA@ZIF-8 crystals (Figure S18). This strongly supports the finding that protein folding is the dominant factor behind the differences observed between BSA and FITC-BSA@ZIF-8 crystals.

### Conclusions

In conclusion, we have demonstrated that the modification of BSA with FITC molecules significantly alters the crystal growth mechanism affecting the encapsulation efficiency, crystal size, and crystal morphology. Circular dichroism studies indicate this is predominantly driven by protein folding within the amorphous precursor phase, and fluorescent spectroscopy studies confirm that proteins remain unfolded in the final MOF crystals. The data also show that different HmIm/Zn ratios will modulate how molecular modification can affect these properties. For example, the effect of modification on the 35:1, 1.25 mg/mL system is that EE% increases from ∼40 to 90%, and the mean crystal diameter increases from 270 ± 50 to 580 ±189 nm. However, the effect of modification for the 4:1, 1.25 mg/mL system is that EE% remains the same (∼100%) while the mean crystal diameter increases from 187 to 1317 nm. These data show that the mechanisms that govern protein EE% and crystal size are at least partially decoupled, which presents a challenge as the role of a protein during crystallization processes is complex. However, these results also present an opportunity to use molecular modifications of proteins to independently tune the structural features and properties of protein@MOFs. Tuning of protein@MOFs requires a deep understanding of nonclassical nucleation pathways and protein folding and aggregation in these pathways. Although each biomolecule will behave differently, we believe that the general mechanisms and tunability with molecular modifications and protein folding should be generalizable to all biomolecules. Future work with other proteins and molecular modifications is needed to confirm this generalizability.

## Methods

### Materials

All chemical reagents used for FITC-BSA@ZIF-8 and BSA@ZIF-8 were obtained from Sigma-Aldrich unless stated otherwise. FITC-BSA was purchased from Sigma-Aldrich post-tagging and purification. Stock solutions of bovine serum albumin, bovine serum albumin fluorescein isothiocyanate, 2-methylimidazole (HmIm), and zinc acetate (Zn) were made using Milli-Q water (ρ > 18 MΩ cm).

### TEM

TEM samples were prepared by pipetting 10× diluted solutions onto TEM grids for ∼ 5–10 min and were then blotted with Kimwipe paper. Further, 400 Mesh Carbon grids were used and purchased from TedPella. Images were obtained using a JOEL-2800 TEM with a Schottky field-type field emission gun at 200 kV in convergent beam mode using a Gatan OneView Camera.

### Cryo-TEM

Cryo-TEM samples were prepared using a Quantifoil R2/2 Holey Carbon Films from Electron Microscopy Sciences or 400 Mesh Carbon grids from TedPella. Prior to sample application, glow discharge was applied to the grids for 70 s. Reaction solutions at various time points were centrifuged for ∼ 2 s, and 3 μL of each sample was taken from the reaction solutions and underwent vitrification using an Automatic Plunge Freezer ME GP2 (Leica Microsystems). Vitrification was performed at a ∼95% humidity with a blot time of 4 s, and samples were plunged into liquid propane. Samples were then analyzed using a JOEL-2100 TEM with a Schottky field-type emission gun set to 200 kV. Images were obtained using Serial EM software or a Gatan OneView Camera.

### SEM

Samples were prepared by pipetting 10 μL of the sample onto 1 mm thick glass slides, which were then coated with 5 nm iridium (Quorum Q150T) to reduce charging. Samples were imaged with a Magellan 400 XRH system with secondary electron images taken at an accelerating voltage ranging from 2 to 3 keV.

### PXRD

After removing all liquid from the top of centrifuged crystal precipitates and allowing samples to air dry, a Rigaku SmartLab X-ray diffractometer was used to obtain PXRD patterns at 40 kV and 44 mA while in Bragg–Brentano mode. Results were plotted with background subtraction using IGOR software.

### In Situ XRD

Samples were initially mixed in glass vials and immediately transferred into 10 mm glass capillaries. Samples were scanned every 10 min for 8 h using a Rigaku Smartlab. The instrument was set to 40 kV and 44 mA and measured in parallel beam/ parallel slit analyzer mode. Results were plotted with background subtraction using IGOR software.

### Fluorescence Microscopy

Fluorescence imaging and microscopy were performed as described in the previous manuscript.^18^ Second harmonic of 960 nm femtosecond pulse radiation (480 nm, 76 MHz, 5 mW) was coupled into an Olympus FluoView 1000 laser scanning microscopy system based on an Olympus IX81 inverted microscope frame. Fluorescence was collected using a 60 × NA = 1.41 oil immersion objective lens (Olympus) in epi geometry. Transmitted light was used for simple morphology mapping and correlation with TEM images. Imaging was performed at various fields of view with resolutions of 800 × 800 and 2048 × 2048 pixels with a scanning speed of 2 μs/pixel. All images were processed to be displayed in RGB (100,0,0) coordinates.

### Mass Spectrometry

MALDI-TOF-MS measurements were performed using a Brûker Ultra Flex Extreme in linear positive mode. Samples were spotted in the water and ran in saturated sinapic acid in a 50:50 water/acetonitrile with 0.1% TFA (trifluoracetic acid). Intact mass measurements were also performed using a Xevo G2-XS Qtof after desalting thru a phenyl–hexyl column BEH guard column. The measurements were performed in positive mode from 400 to 4,000 da. The charge state series were deconvoluted using a Waters’ Masslynx MaxEnt1 algorithm with ranges of 50,000:80,000 g/mol. Baseline subtraction was then performed.

### Circular Dichroism

Circular dichroism samples were diluted to 1 mg/mL using water and were analyzed between 200 and 240 nm in a 10 mm quartz cuvette. Five accumulations for each sample were performed.

### ζ Potential

ζ potential measurements of samples were taken with a Malvern Zetasizer ZS Nano dynamic light scattering instrument. The instrument was set to automatic runs (ranging from 10–100), and triplicate measurements were averaged for each sample. Measurements were performed with samples in a disposable capillary cell from Malvern Panalytical.

## Supporting Information Available

Intrinsic tryptophan fluorescence spectra of proteins and protein@MOFs; PXRD patterns and SEMs of ZIF-8 at varying HmIm/Zn ratios; additional XRD patterns and SEM images of protein@MOFs at protein concentrations of 1.25 and 0.625 mg/mL; details of crystal size analysis methods and additional plots and histograms comparing protein@MOF sizes; low-magnification TEM image of protein@MOFs; FTIR spectra of BSA, ZIF-8, and protein@MOFs; details and controls of the encapsulation efficiency method; details of in situ XRD analysis; methods for particle size analysis; characterization and analysis of partially unfolded BSA in ZIF-8; and additional cryo-TEM and dry-state TEM images of FITC-BSA@ZIF-8 at various time points (PDF)
