# Supplementary material for: Role of Molecular Modification and Protein Folding in the Nucleation and Growth of Protein–Metal–Organic Frameworks
Source: Chem Mater. 2022 Sep 15;34(18):8336–44. doi: 10.1021/acs.chemmater.2c01903 (PMC9523577; doi:10.1021/acs.chemmater.2c01903)
Supplement: Supplementary file 1 — cm2c01903_si_001.pdf [file cm2c01903_si_001.pdf]

Supporting Information: The role of molecular modification and protein folding in the nucleation and growth of protein-metal-organic frameworks

Brooke P. Carpenter[1], A. Rain Talosig[1], Justin Mulvey[2], Jovany Merham[1], Jamie Esquivel[1], Ben Rose[1] , Alana F. Ogata[1], Dmitry A. Fishman[1], Joseph P. Patterson[1,2]*

[1]*Department of Chemistry, University of California, Irvine, Irvine, CA 92697-2025, United States*
[2]*Department of Materials Science and Engineering, University of California, Irvine, Irvine, CA 92697-2025, United States*

Corresponding author email: patters3@uci.edu

Protein Characterization

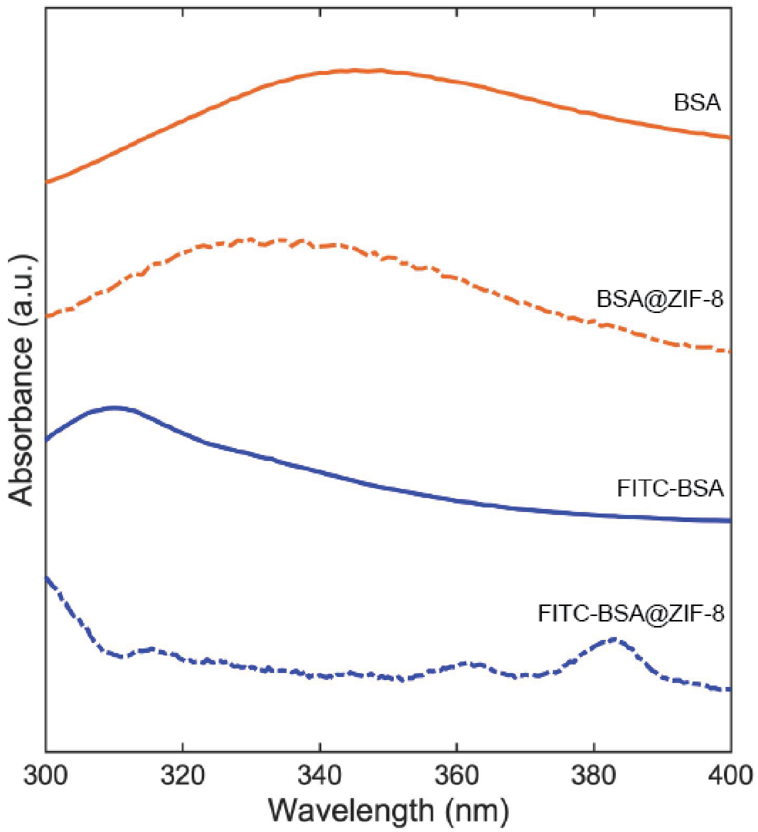

Supplemental Figure S1: Intrinsic tryptophan fluorescence spectra of BSA (orange, solid), BSA@ZIF-8 (orange, dashed), FITC-BSA (blue, solid), and FITC-BSA@ZIF-8. Samples were excited at 280 nm and the emission was analyzed from 300-400 nm.

Crystal structure analysis

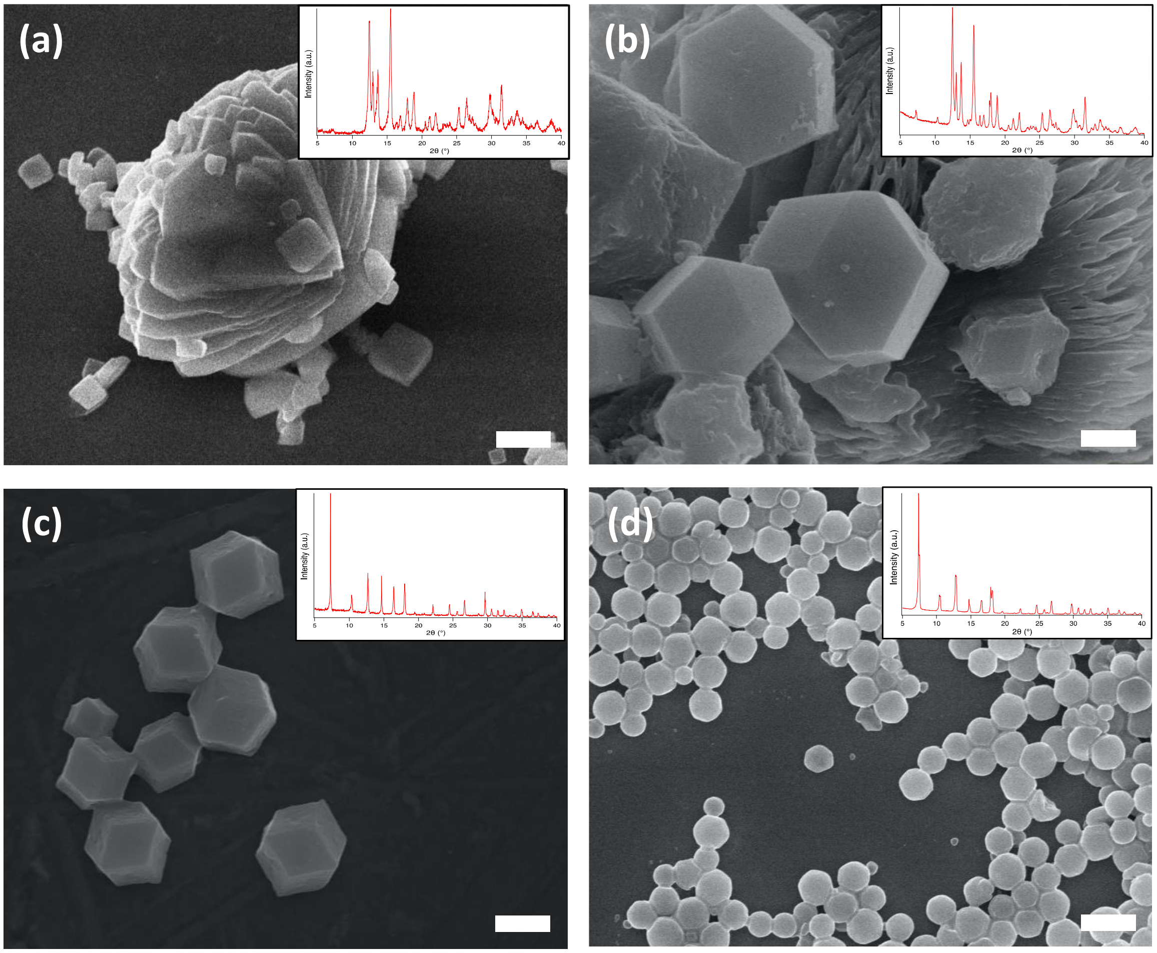

Supplemental Figure S2: SEM images of ZIF-8 at (a) 4:1 (b) 17.5:1 (c) 35:1 (d) 70:1. At 4:1, ZIF-8(dia) is exclusively seen whereas at 17.5:1, a mixture of ZIF-8(dia) and ZIF-8(sod) is seen. At 35:1 and 70:1, only ZIF-8 sod is present. Scale bar is 1 μm.

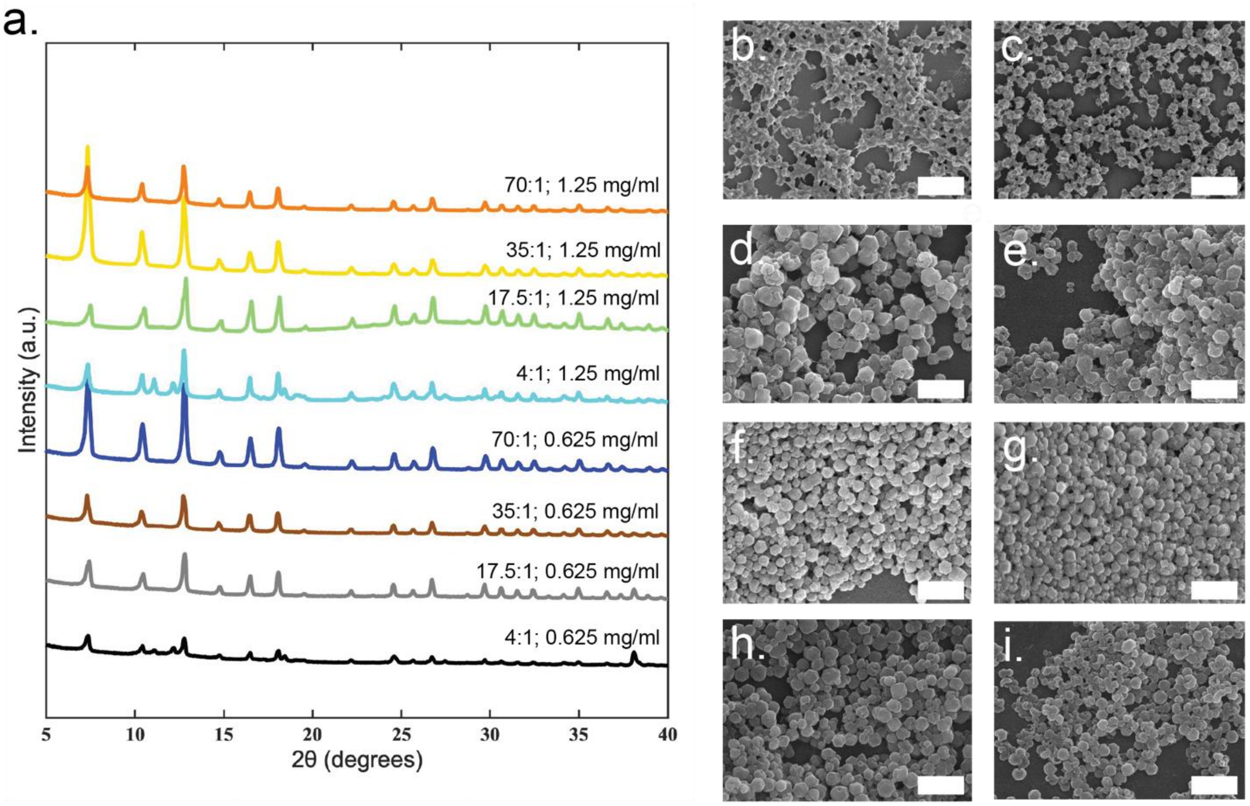

Supplemental Figure S3: (a.) PXRD patterns of BSA@ZIF-8 at varying HmIm:Zn ratios and at final BSA concentrations of either 1.25 mg/ml or 0.625 mg/ml. SEM images of BSA-ZIF-8 with final protein concentrations of 1.25 mg/ml BSA at HmIm:Zn ratios of (b) 4:1 (d) 17.5:1 (f) 35:1 (h) 70:1, and 0.625 mg/ml BSA at HmIm:Zn ratios of (c) 4:1 (e) 17.5:1 (g) 35:1 (i) 70:1. Scale bar is 1 μm.

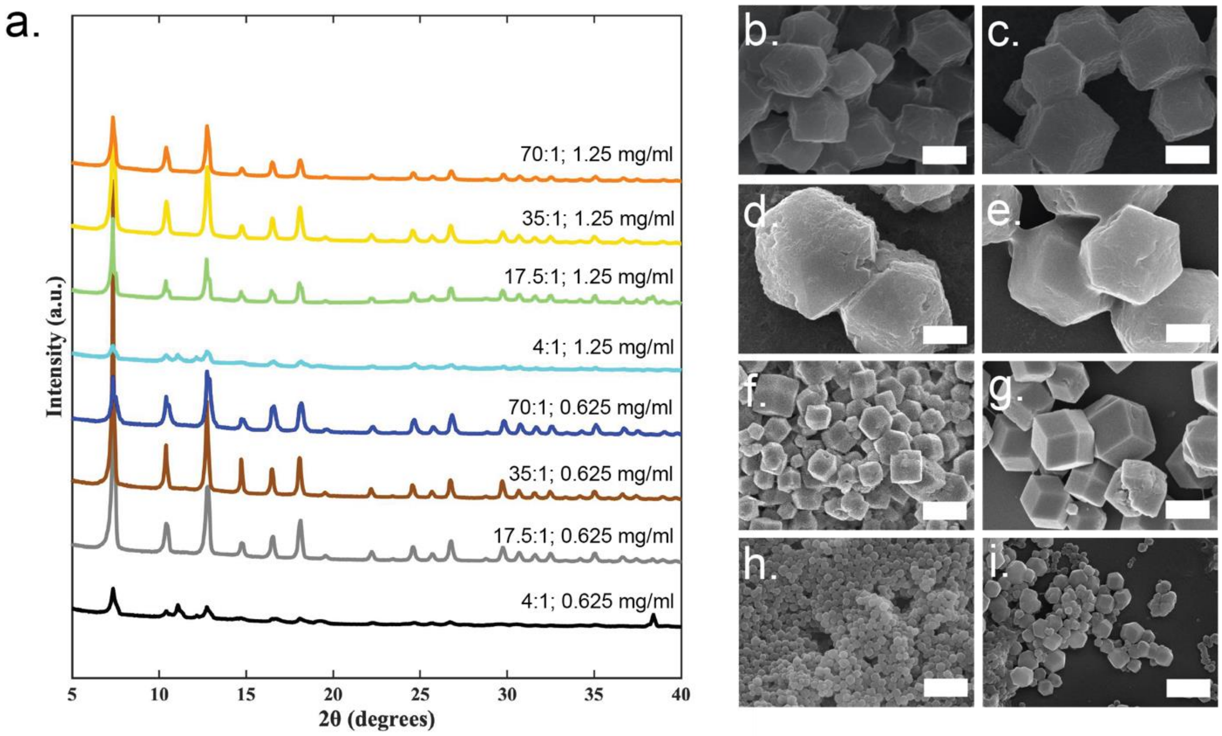

Supplemental Figure S4: (a.) PXRD patterns of FITC-BSA@ZIF-8 at varying HmIm:Zn ratios and at final FITC-BSA concentrations of either 1.25 mg/ml or 0.625 mg/ml. SEM images of FITC-BSA@ZIF-8 crystals with final protein concentrations of 1.25 mg/ml FITC-BSA at HmIm:Zn ratios of (b) 4:1 (d) 17.5:1 (f) 35:1 (h) 70:1, and with final protein concentrations of 0.625 mg/ml FITC-BSA at HmIm:Zn ratios of (c) 4:1 (e) 17.5:1 (g) 35:1 (i) 70:1. Scale bar is 1 μm
.

Crystal Size analysis

SEM was used to determine crystal diameter by averaging the length of ~50-100 crystals per sample using Fiji, ImageJ (Figure S5). From the analysis, crystals sizes were binned into groups of 250 nm and plotted with Prism.

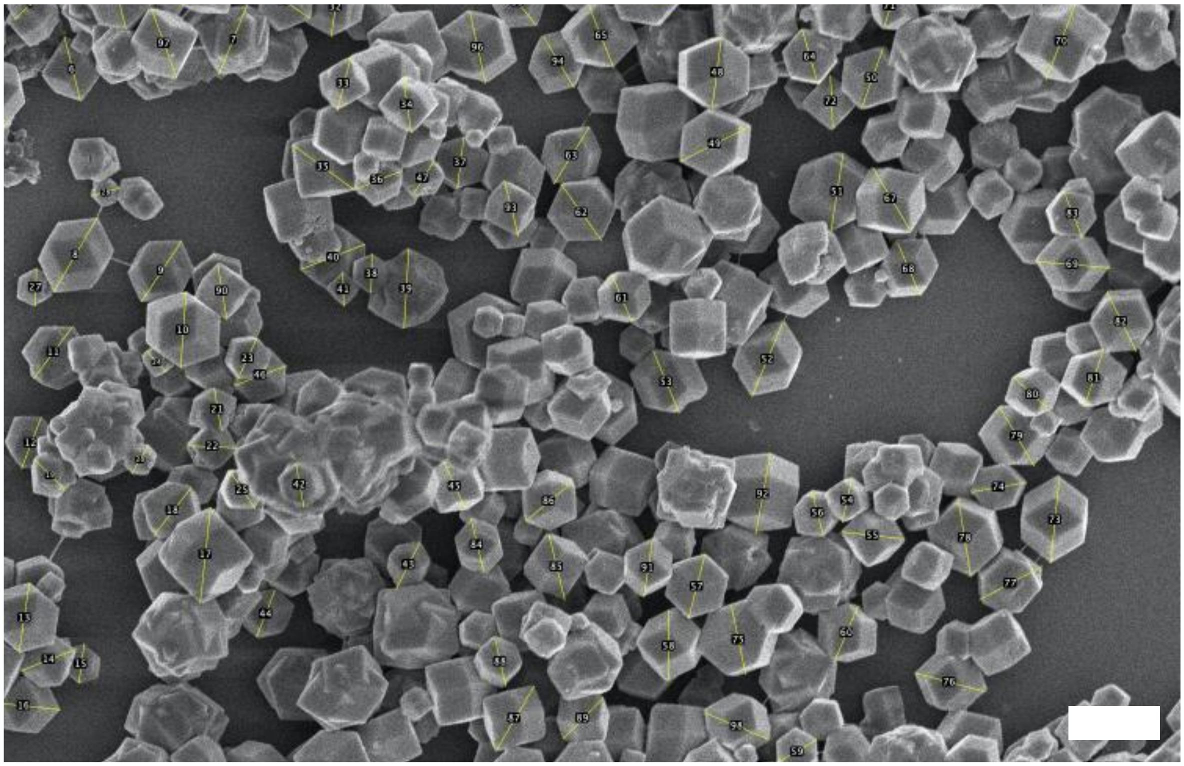

Supplemental Figure S5: SEM image (scale bar 2 μm) of FITC-BSA-ZIF-8 at protein concentration of 0.625 mg/ml at 35 : 1 (HmIm : Zn). Numbered yellow lines indicate the diameter of the crystal measured using Fiji, ImageJ.

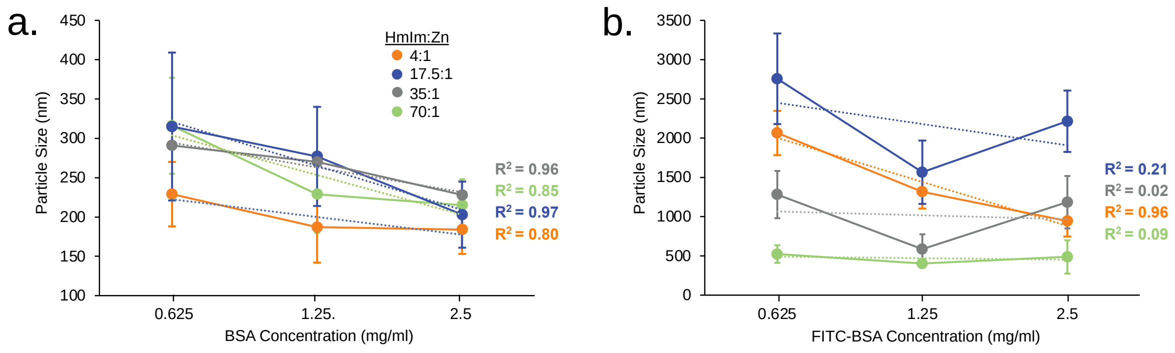

Supplemental Figure S6: Plots of protein concentration versus size (nm) for (a) BSA-ZIF-8 crystals and (b) FITC-BSA-ZIF-8 crystals. The following HmIm:Zn were plotted for each sample: 4:1 (orange), 17.5:1 (blue), 35:1 (grey), 70:1(green). Results demonstrate that for BSA-ZIF-8, the size for each HmIm:Zn decreases with increasing protein concentration. However, no trends could be observed for the FITC-BSA-ZIF-8 systems.

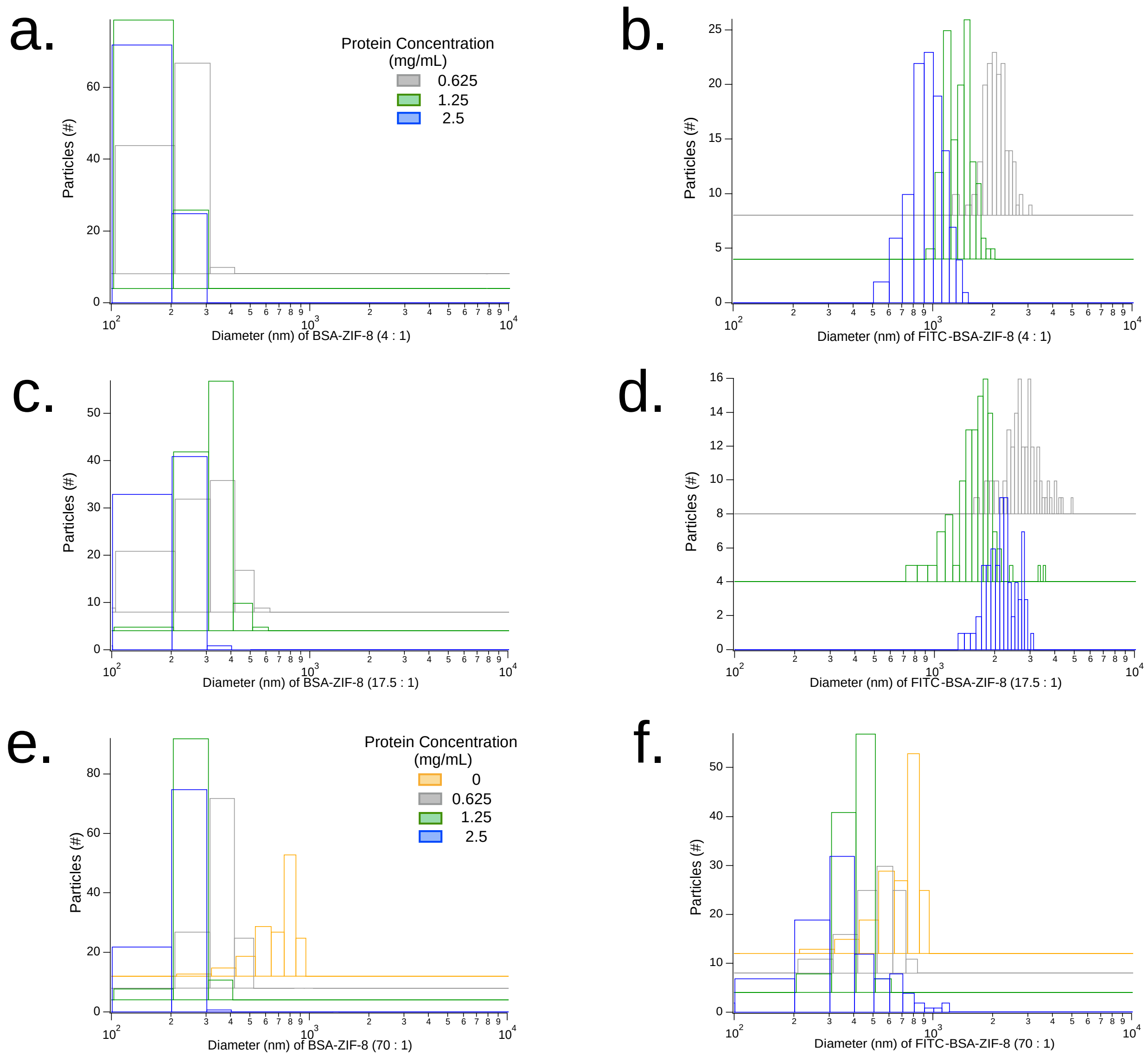

Supplemental Figure S7: Histogram of size measurements from crystals in SEM images of (a) BSA-ZIF-8 at 4:1 (b) FITC-BSA-ZIF8 at 4:1 (c) BSA-ZIF-8 at 17.5:1 (d) FITC-BSA-ZIF-8 at 17.5:1 and (e) BSA-ZIF-8 at 70:1 (f) FITC-BSA-ZIF-8 70:1 using protein concentrations of 0.625 mg/ml (grey), 1.25 mg/ml (green), and 2.5 mg/ml (blue). Yellow bars in 70:1 systems indicate crystals without protein.

Crystal Morphology

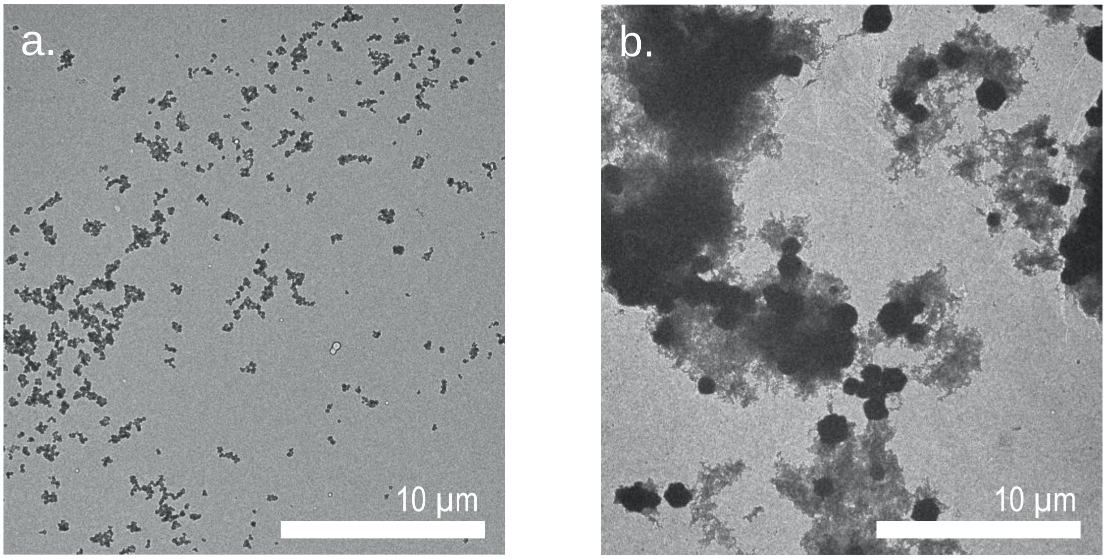

Supplemental Figure S8: TEM images taken of low magnification of (a) BSA-ZIF-8 and (b) FITC-BSA-ZIF-8. The systems were at 35:1 (HmIm:Zn) with final protein concentrations of 2.5 mg/ml. Samples were washed 3x in water, 1x in methanol, and diluted 10x in methanol Images were taken at low magnification to capture a broad area of sample and validate that our findings are consistent throughout the sample.

Protein Incorporation and Encapsulation Efficiency

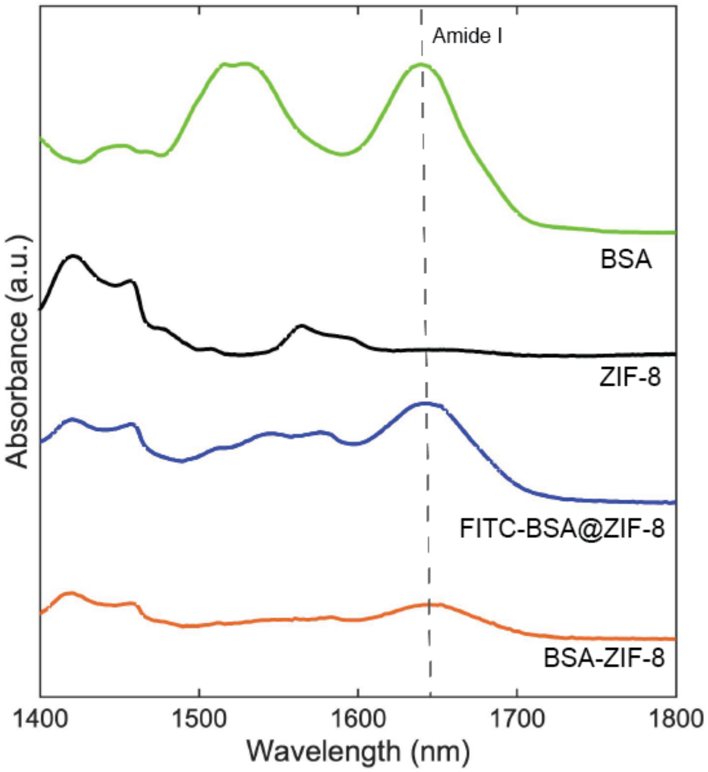

Supplemental Figure S9: FTIR spectra of BSA(green), ZIF-8 (black), FITC-BSA@ZIF-8 (blue), and BSA@ZIF-8 (orange). The protein@MOFs were centrifuged for 10 minutes at 10,000 rpm and washed with water 3x. Samples washed with water were compared to samples that were washed with an additional time with 1x SDS buffer, but little to no difference could be observed in the protein@MOF spectra.

Intrinsic Tryptophan Fluorescence Controls: When measuring intrinsic tryptophan fluorescence of a MOF supernatant, excess HmIm and Zinc ions are likely in solution coordinating with remaining protein. Such coordination would alter the fluorescent intensity compared to the isolated protein. HmIm can influence fluorescence intensity based on two factors: pH and BSA/HmIm interactions.[1,2] When dissolved in water, HmIm alters the pH by making it more basic due to HmIm having a pka ~8.[3] This change in the pH for the protein environment is not desirable since the protein can undergo conformational change causing varying fluorescent intensities. However, diluting the supernatants in phosphate buffer (pH 6.7) alleviates this change in pH, assuring that BSA is in the same protein conformation for each measurement.

To address the influence of BSA/HmIm interactions, fluorescent controls were made using each protein/HmIm condition for the 4:1, 17.5:1, 35:1, and 70:1 systems, excluding the zinc. For each control, BSA (10 mg/ml 5mg/ml, 2.5 mg/ml, 1.25 mg/ml, 0.625 mg/ml, 500 uL), HmIm (5600 mM, 2800 mM, 1400 mM, and 3200 mM, 500 uL, and water (1 ml) were combined and then diluted by 10 in phosphate buffer to give final final protein concentrations of 0.25 mg/ml, 0.125 mg/ml, 0.0625 mg/ml, 0.312 mg/ml and 0.0156 mg/ml and final HmIm concentrations of 140 mM,

70 mM, and 35 mM and 8 mM. Each system was measured by exciting at 280 nm and measuring the emission at 340 nm. It was reported that as the HmIm concentration decreases, the slope of the curve increases (Figure S10). The encapsulation efficiencies for each system were calculated using each calibration curve – one with protein and HmIm and the other with protein only. However, the results between the two calculations only varied by 0-8% due to low protein concentrations in supernatant resulting in lower intensities. For each of the calibration curves, with and without HmIm, the lower protein concentration ranges appear very close together, which explains the similar EE% calculated with both curves. This method potentially would not have worked as well if higher protein concentrations/intensities had been recorded for the supernatants. Thus, the EE% were recorded in the main text based on the protein only calibration curve.

To prevent protein-zinc binding interactions from altering the fluorescent measurements, EDTA was added to sequester zinc ions. Controls were made by first incubating BSA and zinc together in a solution containing BSA (2.5 mg/ml, 500 uL), zinc acetate (40 mM), and water (500 uL). Once incubated for ~30 min, 0.2 uL of the solution was added to separate vials containing 2.8 mL of phosphate buffer with various concentrations of EDTA (0 mM, 10 mM, 20 mM, 45 mM, and 90 mM). It was found that at EDTA concentrations greater than or equal to 20 mM, the fluorescent intensity plateaus (Figure S11a). Upon addition of BSA (2.5 mg/ml, 0.5 mL), Zinc Acetate (40 mM, 1 mL), and water (0.5 mL), the solution becomes turbid due formation of BSA/Zinc aggregates (Figure S11b). Upon addition of 90 mM EDTA, the solution becomes clear due to the metal chelator sequestering the metal ions from BSA.

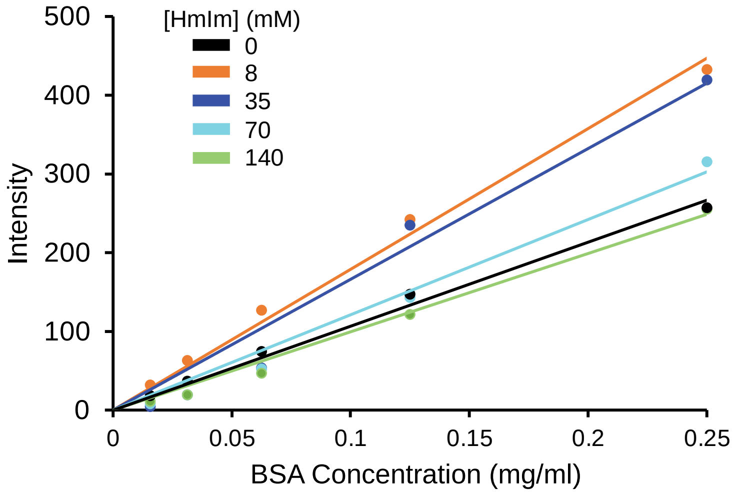

Supplemental Figure S10: HmIm fluorescent control using protein concentrations of 0.25 mg/ml, 0.125 mg/ml, 0.0625 mg/ml, 0.312 mg/ml and 0.0156 mg/ml with HmIm concentrations of 0 mM (black), 8 mM (orange), 35 mM (yellow), 70 mM (blue), and 140 mM (green).

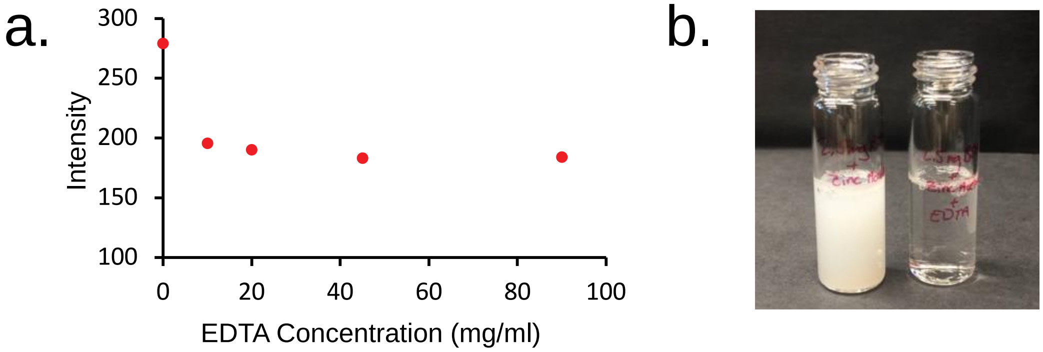

Supplemental Figure S11: (a) Fluorescent measurements of addition of EDTA (0 mM, 10 mM, 20 mM, 45 mM, and 90 mM, 2.8 mL) to solution of BSA/Zinc solution (0.2 mL). (b). Image of BSA/Zinc solution before (left) and after (right) addition of EDTA (90 mM).

Calibration Curves: A BSA calibration curve was made using the following protein concentrations in phosphate buffer (pH 6.7) with 80 mM EDTA: 0.25 mg/ml, 0.125 mg/ml, 0.0625 mg/ml, 0.03125 mg/ml, and 0.0156 mg/ml. Triplicates of each system were made and measured with Cary Eclipse Spectrophotometer using an excitation of 280 nm and emission of 340 nm (Figure S12a). Supernatants of each MOF system were diluted by 10-fold in the Phosphate/EDTA solution and compared to the standard calibration curve.

FITC-BSA calibration curves were made using the following protein concentrations: 0.025 mg/ml, 0.0125 mg/ml, 0.00625 mg/ml, 0.003125 mg/ml, and 0.00156 mg/ml. Triplicates of each concentration were made and measured using an excitation of 494 nm and emission of 520 nm (Figure S12b). Supernatants of FITC-BSA-ZIF-8 systems were diluted by 100-fold in phosphate buffer (pH 6.7) and compared to the standard calibration curve.

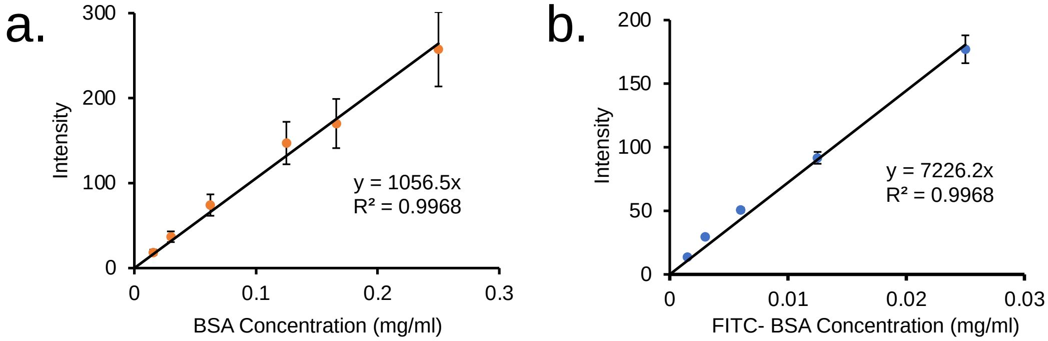

Supplemental Figure S12: Tryptophan fluorescence standard calibration curve for (a) BSA and (b) FITC-BSA. Triplicate measurements of separate protein stocks were taken for each protein concentration and averaged. Each data point represents an average of three runs with the error bars indicating the standard deviation of the three runs.

Bradford Assay: To validate the EE% results from intrinsic tryptophan fluorescence, the Bradford reagent is utilized. The Bradford reagent used was a ready-made solution from Sigma Aldrich containing Coomassie Blue G-250. To make the standard calibration curve, Bradford reagent (3 mL) was added to 100 µL of protein solution (0.1mg/mL, 0.425 mg/mL, 0.75 mg/mL, 1.05 mg/mL, and 1.4 mg/mL) and inverted gently to mix. The samples were incubated at room temperature for 10 minutes. In disposable cuvettes, the absorbances of the protein samples were taken at 595 nm using UV-Vis on a Nanodrop 2000C (Figure S4). The supernatants (100 µL) from BSA -ZIF-8 systems (4:1, 17.5: 1, 35:1, and 70:1) were then mixed with Bradford reagent (3 mL)) and measured with absorbance. EE% were calculated based on the standard calibration (Figure S13). Results from the measurements were then compared to the fluorescent method (Figure S14).

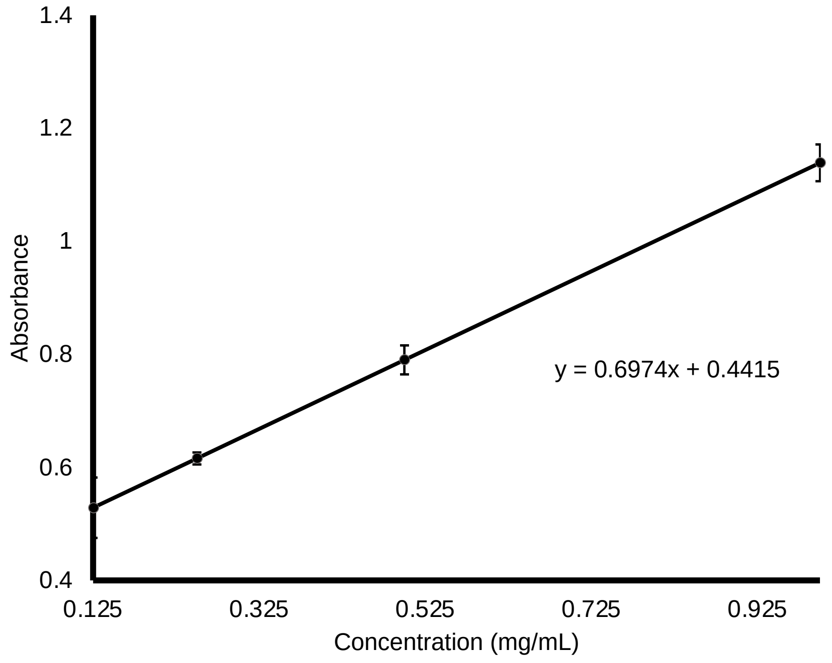

Supplemental Figure S13: Bradford assay standard calibration curve for BSA.

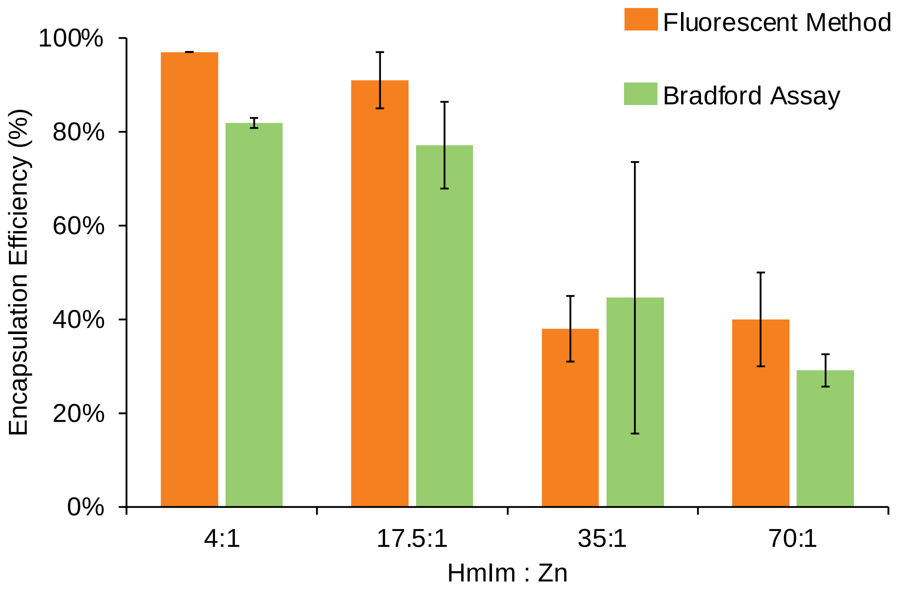

Supplemental Figure S14: EE% method comparison of protein@MOF supernatants using the Bradford assay (orange) and the fluorescent method (blue).

In situ XRD analysis:
Instantaneous peaks or valleys can be caused by low signal to noise, so a sliding-window Gaussian weighted mean was applied to each XRD region where the mean signal was used to smooth the data (eq 1). A standard deviation of 3 data points was used. The same trends can be seen in both the raw and smoothed data shown in Figure S15.

$$S_s = \text{signal convolution}\,(S_r, k_{gauss}) \qquad (1)$$

where S_s: Gaussian smoothed mean signal
S_r: Raw mean signal
k_{gauss}: 1D normalized Gaussian kernel

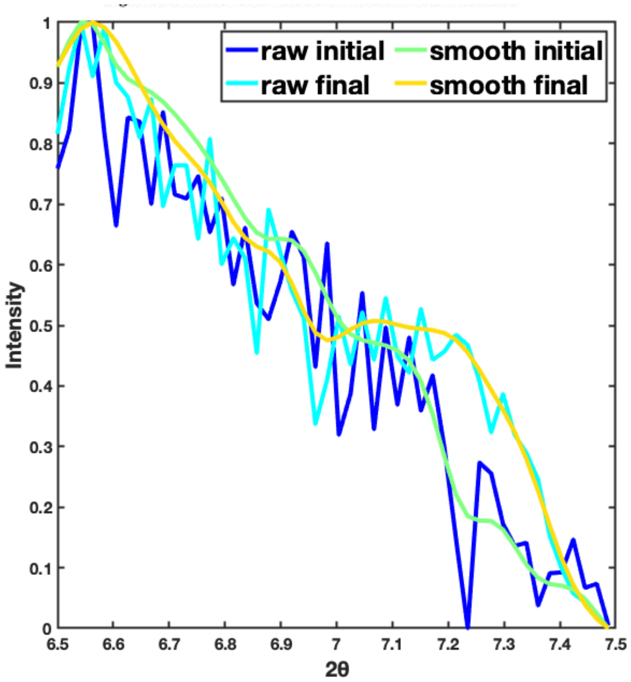

Supplemental Figure S15: In-situ XRD Data of FITC-BSA@ZIF-8 at initial (0 mins) and final (450 minutes) timepoints. Raw data has been plotted for the intial (purple) and final (blue) XRD patterns. Smoothed data has also been plotted for the initial (green) and final (yellow) XRD patterns.

Particle Size Analysis
Particles were manually picked and overlaid using the same method as Ogata et.al. [4] The sizes of the particles were then calculated using full-width-half-max (FWHM) algorithm to determine the size between multiple particles in a consistent manner. The FWHM is different from our previous approach (ref 4) where we estimate both the core diameter and shell size manually.

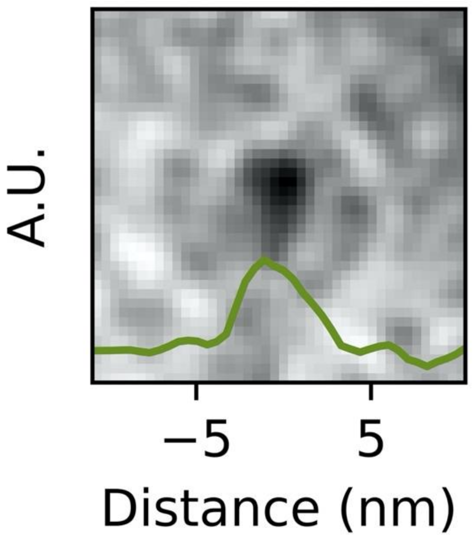

Supplemental Figure S16: Size Analysis of particles in cryoTEM image at 1hr of 35:1 FITC-BSA@ZIF-8 with final protein concentration of 2.5 mg/ml.

Partially Unfolded Protein Analysis

BSA was unfolded by aging a 10 mg/ml BSA solution in water at room temperature for 6 months.

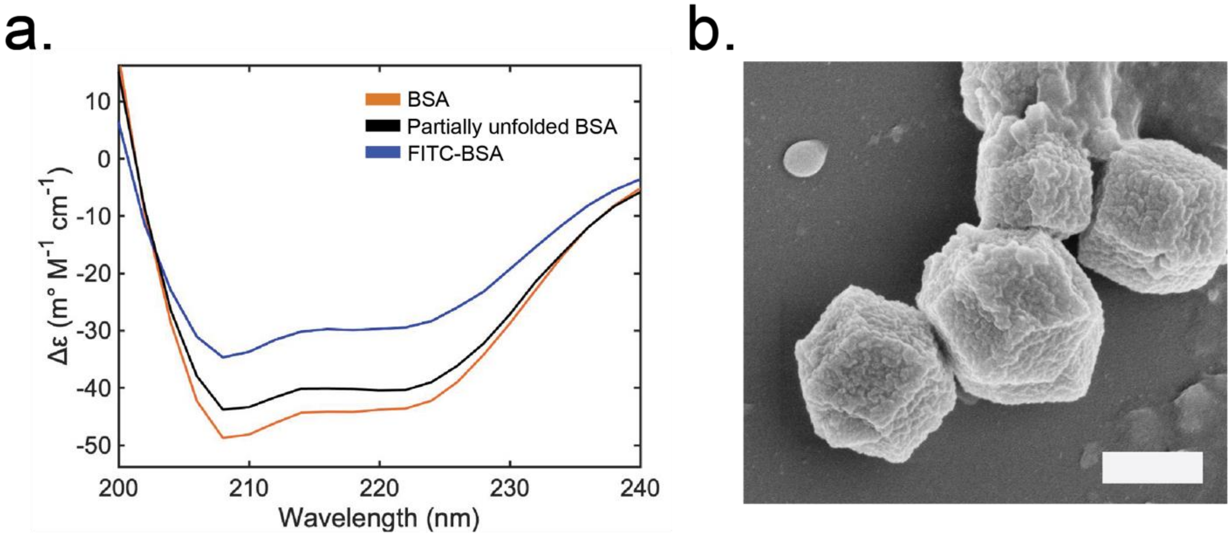

Supplemental Figure S17: a.) Circular dichroism of BSA (orange), partially unfolded BSA (black), and FITC-BSA (blue). SEM image of partially unfolded BSA encapsulated in 35:1 ZIF-8 at final protein concentrations of 2.5 mg/ml. Scale bar is 500 nm.

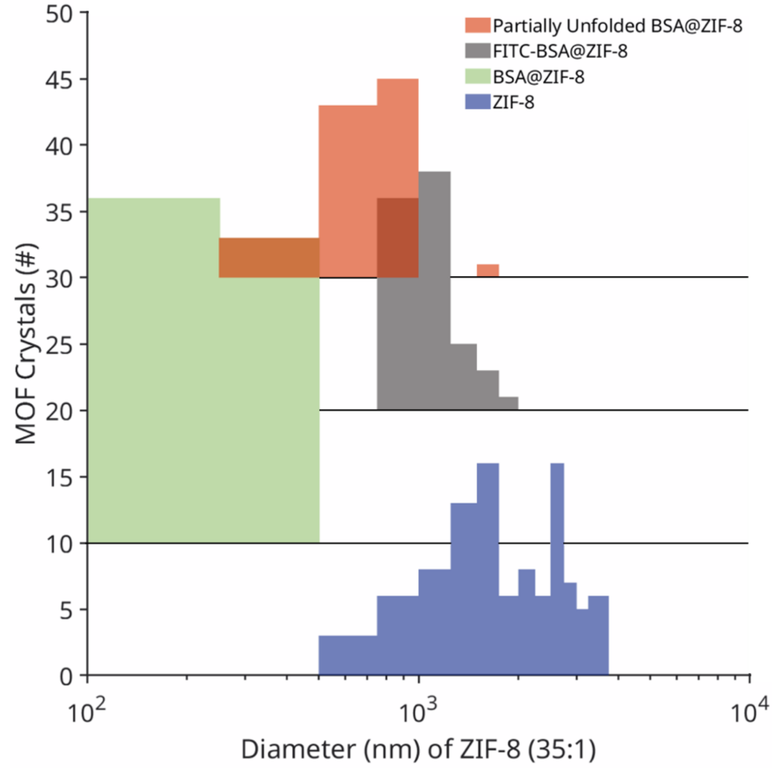

Supplemental Figure S18: Size histogram of the particle sizes of partially unfolded BSA in ZIF-8 (orange), FITC-BSA-ZIF-8 (gray), BSA@ZIF-8 (green), and ZIF-8 (blue). MOFs were synthesized at HmIm:Zn ratio of 35:1 with a final protein concentrations of 2.5 mg/ml.

Additional TEM Images

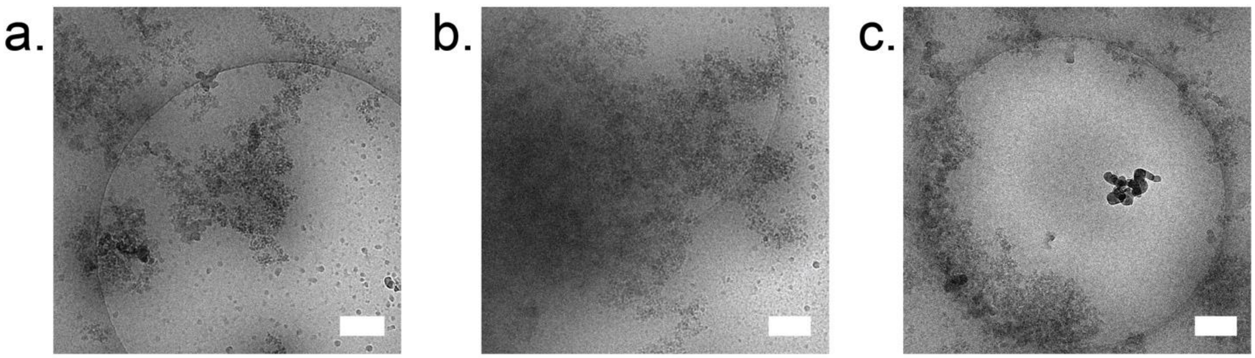

Supplemental Figure S19: CryoTEM images of FITC-BSA@ZIF-8 at 1 minute when at HmIm:Zn ratio of 35:1 and final protein concentration of 2.5 mg/ml. The scale bars for (a.) and (b.) are 100 nm, and the scale bar for (c.) is 200 nm.

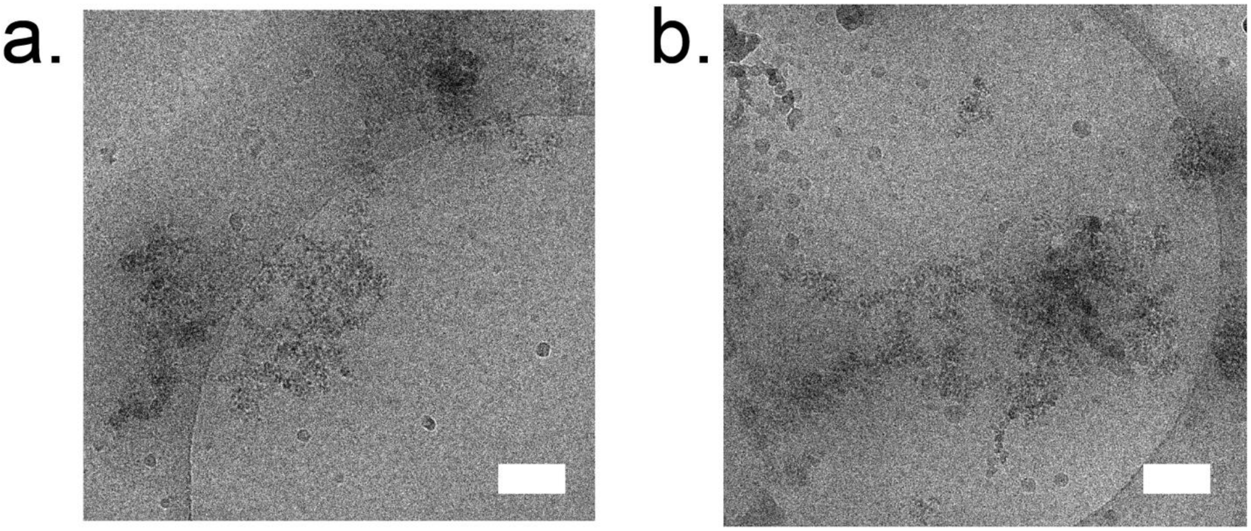

Supplemental Figure S20: CryoTEM images of FITC-BSA@ZIF-8 at 5 minutes when at HmIm:Zn ratio of 35:1 and final protein concentration of 2.5 mg/ml. The scale bar is 100 nm.

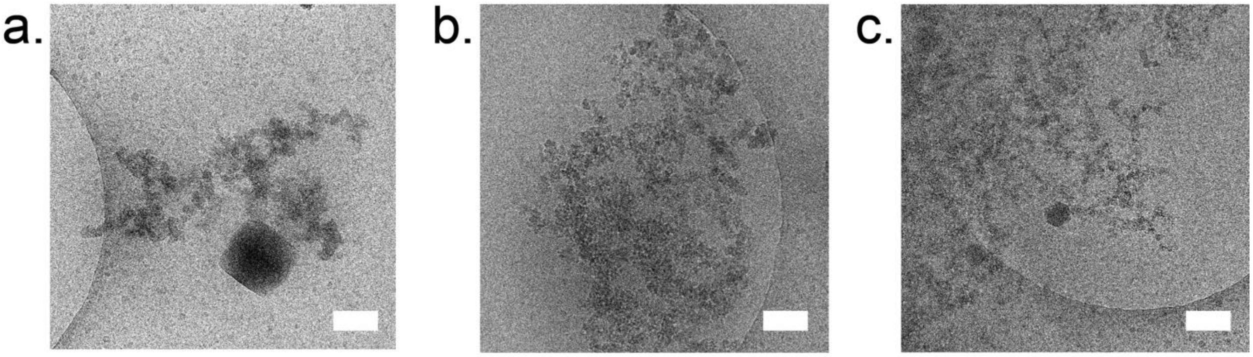

Supplemental Figure S21: CryoTEM images of FITC-BSA@ZIF-8 at 30 minutes when at HmIm:Zn ratio of 35:1 and final protein concentration of 2.5 mg/ml. The scale bar is 100 nm.

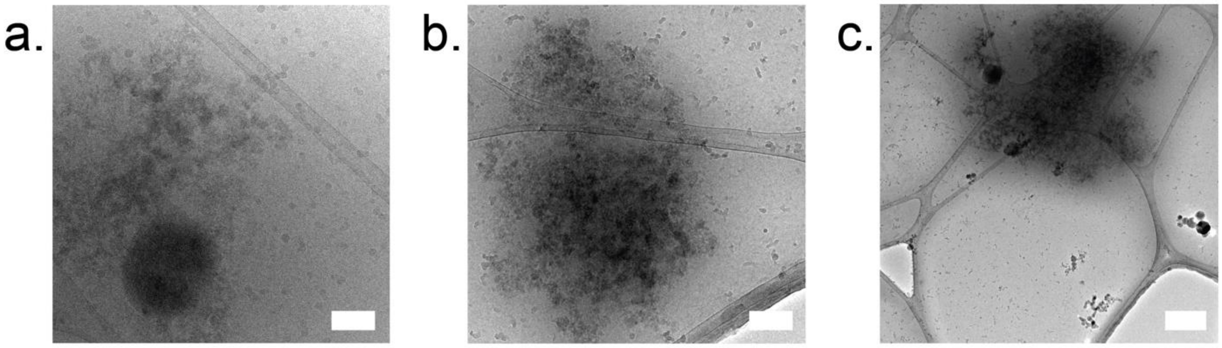

Supplemental Figure S22: CryoTEM images of FITC-BSA@ZIF-8 at 1 hour when at HmIm:Zn ratio of 35:1 and final protein concentration of 2.5 mg/ml. The scale bars for (a.) and (b.) are at 100 n,m and the scale bar for (c.) is at 1 μm.

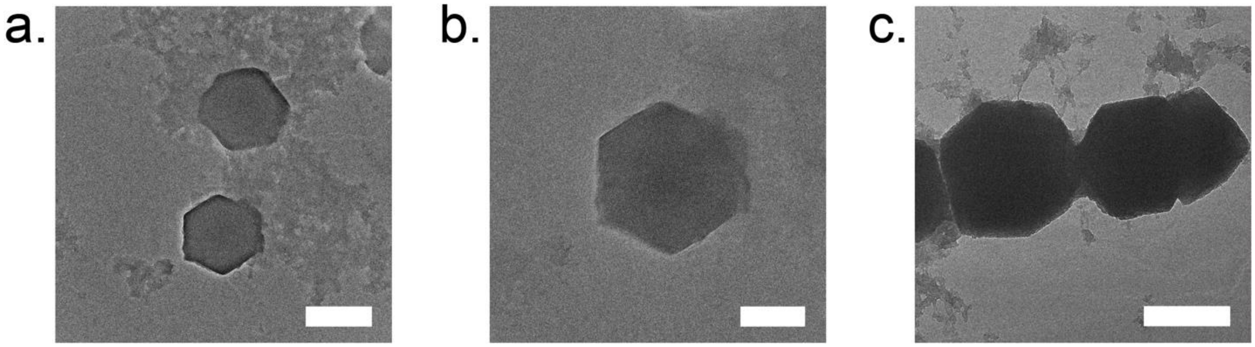

Supplemental Figure S23: Dry state TEM of FITC-BSA@ZIF-8 after 24-hour synthesis and 3x washes with water. Scale bar for (a.) is 500 nm, and the scale bar for (b.) and (c.) is at 1 μm.

References:

(1) Liao, S.-M.; Du, Q.-S.; Meng, J.-Z.; Pang, Z.-W.; Huang, R.-B. The Multiple Roles of Histidine in Protein Interactions. *Chem Cent J* **2013**, *7*, 44. https://doi.org/10.1186/1752-153X-7-44.

(2) Guckeisen, T.; Hosseinpour, S.; Peukert, W. Effect of PH and Urea on the Proteins Secondary Structure at the Water/Air Interface and in Solution. *Journal of Colloid and Interface Science* **2021**, *590*, 38–49. https://doi.org/10.1016/j.jcis.2021.01.015.

(3) Lenarcik, B.; Ojczenasz, P. The Influence of the Size and Position of the Alkyl Groups in Alkylimidazole Molecules on Their Acid-Base Properties. *Journal of Heterocyclic Chemistry* **2002**, *39* (2), 287–290. https://doi.org/10.1002/jhet.5570390206.

(4) Ogata, A. F.; Rakowski, A. M.; Carpenter, B. P.; Fishman, D. A.; Merham, J. G.; Hurst, P. J.; Patterson, J. P. Direct Observation of Amorphous Precursor Phases in the Nucleation of

Protein–Metal–Organic Frameworks. *J. Am. Chem. Soc.* **2020**, *142* (3), 1433–1442. https://doi.org/10.1021/jacs.9b11371.
